# Anti–Zika Virus Activity and Isolation of Flavonoids from Ethanol Extracts of *Curatella americana* L. Leaves

**DOI:** 10.3390/molecules28062546

**Published:** 2023-03-10

**Authors:** Lienne D’Auria Lima, Adriana Cotta Cardoso Reis, Jordano Augusto Carvalho Sousa, Gabriel Mendonça Valente, Breno de Mello Silva, Cíntia Lopes de Brito Magalhães, Markus Kohlhoff, Luiz Fernando de Medeiros Teixeira, Geraldo Célio Brandão

**Affiliations:** 1 Departamento de Farmácia, Escola de Farmácia, Universidade Federal de Ouro Preto, Campus Morro do Cruzeiro, Ouro Preto 35.400-000, MG, Brazil; 2 Departamento de Ciências Biológicas, Instituto de Ciências Exatas e Biológicas, Universidade Federal de Ouro Preto, Campus Morro do Cruzeiro, Ouro Preto 35.400-000, MG, Brazil; 3 Laboratório de Química de Produtos Naturais Bioativos, Fundação Oswaldo Cruz. Instituto René Rachou, Belo Horizonte 30.190-009, MG, Brazil; 4 Departamento de Análises Clínicas, Escola de Farmácia, Universidade Federal de Ouro Preto, Campus Morro do Cruzeiro, Ouro Preto 35.400-000, MG, Brazil

**Keywords:** *Curatella americana*, Zika virus, cytotoxicity, phytochemistry, flavonoid

## Abstract

The ethnomedicinal plant *Curatella americana* L. (Dilleniaceae) is a common shrub in the Brazilian Cerrado, whose ethanolic extract showed significant in vitro anti–Zika virus activity by the MTT colorimetric method. Currently, there is no drug in clinical use specifically for the treatment of this virus; therefore, in this work, the antiviral and cytotoxic properties of the ethanolic extract, fractions, and compounds were evaluated. The ethanolic extract of the leaves showed no cytotoxicity for the human MRC-5 cell and was moderately cytotoxic for the Vero cell (CC_50_ 161.5 ± 2.01 µg/mL). This extract inhibited the Zika virus multiplication cycle with an EC_50_ of 85.2 ± 1.65 µg/mL. This extract was fractionated using the liquid–liquid partition technique, and the ethyl acetate fraction showed significant activity against the Zika virus with an EC_50_ of 40.7 ± 2.33 µg/mL. From the ethyl acetate fraction, the flavonoids quercetin-3-*O*-hexosylgallate (**1**), quercetin-3-*O*-glucoside (**2**), and quercetin (**5**) were isolated, and in addition to these compounds, a mixture of quercetin-3-*O*-rhamnoside (**3**) and quercetin-3-*O*-arabinoside (**4**) was also obtained. The isolated compounds quercetin and quercetin-3-O-hexosylgallate inhibited the viral cytopathic effect at an EC_50_ of 18.6 ± 2.8 and 152.8 ± 2.0, respectively. Additionally, analyses by liquid chromatography coupled to a mass spectrometer allowed the identification of another 24 minor phenolic constituents present in the ethanolic extract and in the ethyl acetate fraction of this species.

## 1. Introduction

Zika virus (ZIKV) is an arbovirus belonging to the family Flaviviridae, genus *Flavivirus*, and it is transmitted by mosquitoes of the genus *Aedes*. The virus was first isolated in 1947 in Uganda, Africa, and by 2007, very few human cases were described, all of which were associated with mild clinical symptoms [1]. However, since 2007, ZIKV has spread explosively and unexpectedly, infecting millions of people worldwide and in some cases even causing death [2,3].

ZIKV is believed to have led to a global crisis due to its unexpected links with testicular damage [4], eye damage [5], Guillain–Barré syndrome, fetal microcephaly [6,7], and potentially other neural complications. This emerging pathogen is an RNA virus, and although ZIKV is an arthropod-borne virus, transmission through sexual contact has also been reported [8].

Published results have shown that four different platforms are being used to develop ZIKV vaccines. While there are strong candidates for ZIKV vaccines, which protected rats and *Rhesus monkeys* from lethal ZIKV changes, their safety and cost issues still need to be addressed before becoming licensable vaccines that have a significant impact on overall health [9,10,11].

However, to date, there is no approved vaccine against Zika, and there is an imminent risk of the virus emerging and re-emerging in several countries around the world due to the prevalence of vectors and the high volumes of trade and travel [12], as well as considering the severe symptoms, such as Zika fever, microcephaly, birth defects, and Guillain–Barré syndrome [13].

Antiviral treatments against ZIKV are therefore necessary not only to end ZIKV-associated morbidities but also to disrupt the chain of transmission. Some broad-spectrum antivirals, such as interferons, ribavirin, and favipiravir, are not suitable for use against ZIKV as they may be harmful to pregnant women [14].

Despite continuous progress in the development of new antivirals, viral infections are still one of the leading causes of death worldwide. These infections can be controlled through either prophylactic or curative therapeutic measures. However, since viruses are metabolically inert particles, they need the metabolic pathways of living host cells to multiply, which makes it difficult to design efficient treatment modalities without causing damage to host cells [15,16]. Thus, considerable efforts have been made to find antiviral substances with satisfactory therapeutic effects and/or with new mechanisms of action, with higher plants presenting an excellent potential for prospecting for antiviral substances. The knowledge that plants have a variety of chemical constituents capable of inhibiting the cytopathic effect of viruses with different multiplication cycles makes it important to research natural products with this activity [16]. Our research group has shown that several species rich in phenolic compounds, such as flavonoids, are promising sources of substances with anti-ZIKV activity [17,18,19,20,21].

The phytochemical study of medicinal plants is a strategic alternative in the search for new therapeutic agents. The bibliographic survey and popular knowledge serve as the basis for identifying the pharmacological activity of medicinal plants. In the Brazilian Cerrado, there is great diversity of species and environments, making this biome have a high potential for use [22,23].

One of the highlights of the Cerrado due to its abundance is the species *Curatella americana*, popularly known as lixeira, cajueiro-bravo, or sambaíba, which is the only representative of the genus *Curatella* found in Brazil [24]. The species was identified by [25] as having a high value of ecological importance, when compared with other 63 species in the Cerrado strict sense area. It has a wide distribution with some penetration in the Amazon and Pantanal [26].

The beneficial effects of *C. americana* have been described in scientific research, and it is indicated by its popular use. The anti-inflammatory, analgesic, antihypertensive, hypolipidemic, antimicrobial, antioxidant, antileishmanial, antiulcerogenic, antifungal, and vasodilatory effects of the hydroethanolic extract of stems and leaves of *Curatella americana* L. were evaluated [24,27,28,29,30,31,32].

Despite being an important species of the Cerrado biome, there are relatively few chemical studies of extracts from this plant for medical use. El-Azizi et al. reported the isolation and identification of the flavonol glycoside avicularin and gallic acid [33]. Recent work using liquid chromatography techniques coupled to mass detectors identified a series of phenolic compounds, such as kaempferol, quercetin, and their glycosylated derivatives [34,35].

In this study, we carried out a survey of potential natural antiviral products belonging to plants native to the Cerrado biome and extracts from the leaves of *C. americana*, which showed very promising anti–Zika virus activity. Thus, this species was chosen for a bioguided phytochemical investigation due to its antiviral activity, resulting in the isolation of five flavonols. Additionally, we performed analyses using liquid chromatography coupled to a mass spectrometer to identify some minor constituents.

## 2. Results and Discussion

**Figure 1:**
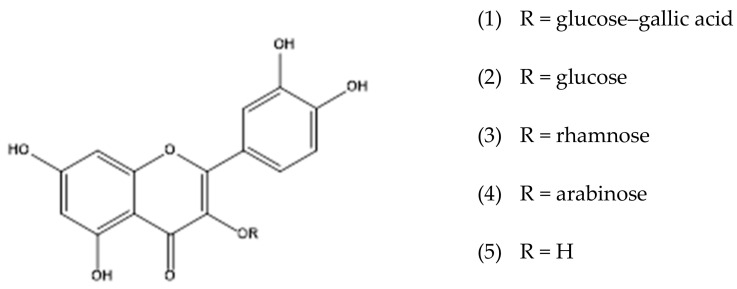
Chemical structures of flavonoids from the leaves of *Curatella americana*.

Extracts obtained from *C. americana* have been studied in different biological models [28,29,30,31,32]. However, few works report the isolation of chemical constituents of this plant species. In this biography study, three flavonoids were isolated, including quercetin (**5**), quercetin-3-*O*-glucoside (**2**), and quercetin-3-*O*-hexosylgallate (**1**). Additionally, a mixture containing quercetin-3-*O*-rhamnoside (**3**) and quercetin-3-*O*-arabinoside (**4**) was also obtained. The structures of the isolated compounds are shown in Figure 1 All isolated compounds were subjected to analysis by ^1^D and ^2^D NMR spectra shown in the Supplementary Material Section (Figures S1–S29). In these spectra, it is possible to observe that the carbons located in position 3 of the flavonic genin have a chemical shift (δ) between 135.0 and 137.0 ppm. This is characteristic of oxygenated flavonoids at this position. Signs of hydrogenated carbons with chemical shifts compatible with positions 6 and 8 of genin (δ 94.0 and 100.0 ppm, respectively) are also observed. Additionally, signals are observed in more shielded regions of the spectra, attributed to sugar residues. All ^13^C and ^1^H NMR data are described in the Materials and Methods section. The data obtained from the NMR analyses suggest that it is a series of oxygenated flavonoids at carbon 3. The structure confirmation of the isolated compounds was obtained by analyzing the UPLC–DAD–HRMS data, and with these data, it was possible to obtain the molar mass of each compound and also the UV data with bands I and II; thus, that exhibits maximum absorption characteristic of flavonoids (Table 1).

**Figure 2:**
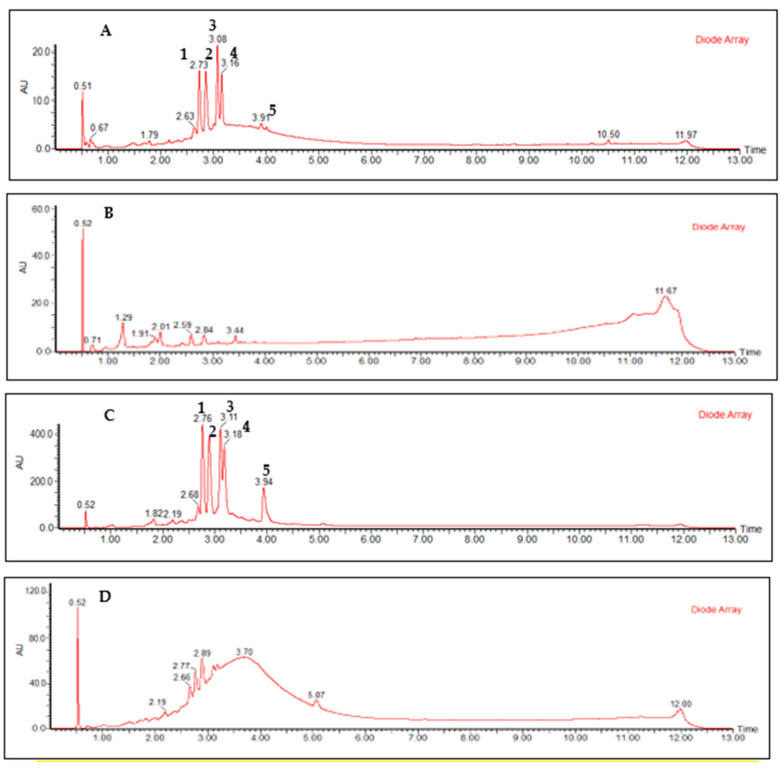
RP-UPLC-DAD profile of ethanolic extract (**A**), dichloromethane fraction (**B**), ethyl acetate fraction (**C**), and aqueous fraction (**D**) of *Curatella americana* leaves. Conditions: CHS130 100 RP-18 column (1.7 μm, 50 × 3 mm i.d.). Elution was carried out with a linear gradient of water with 0.1% formic acid and acetonitrile with 0.1% formic acid (from 5% to 95% of acetonitrile with 0.1% formic acid in 11 min), and the UPLC fingerprints were registered on an Acquity (Waters, Milford, Massachusetts, USA) apparatus with a UV-DAD detector (Waters 2996). (**1**) Quercetin-3-*O*-hexosylgallate, (**2**) quercetin-3-*O*-glucoside, (**3**) quercetin-3-*O*-rhamnoside, (**4**) quercetin-3-*O*-arabinoside, and (**5**) quercetin.

Figure 2 shows the chromatographic profiles of the leaf extract and the fractions obtained in the liquid–liquid partition. The qualitative analysis of these chromatographic profiles suggests that the process employed was efficient in concentrating the flavonoids in the ethyl acetate fraction.

The results are in agreement with the literature reported for the chemistry of this plant species from which quercetin-3-*O*-arabinoside and gallic acid have already been isolated [33]. Recent work analyzing leaf extracts of the species by UPLC–MS also identified flavonoids as major constituents [34,35].

In order to identify minor phenolic constituents from the ethanolic extract and ethyl acetate fraction active in antiviral assays, analyses using UPLC–MS and MS/MS were performed. The data obtained in these analyses are compiled in Table 1. After a careful analysis of the data, it was possible to identify 24 phenolic compounds in addition to the compounds already isolated. These results once again demonstrate that extracts from this plant species are promising sources for obtaining flavonoids.

The cytotoxicity of ethanolic extracts of stems, leaves, fractions, and substances isolated from the *C. americana* species was evaluated by the colorimetric MTT method in Vero cell lines (derived from African green monkey kidneys) and MRC-5 (derived from normal human lung tissue). The results of CC_50_ are described in Table 2.

According to the literature, crude extracts whose CC_50_ ≤ 20 μg/mL are classified as extracts with high cytotoxic effect [36], and those extracts with CC_50_ > 100.0 μg/mL are not considered cytotoxic [37]. Thus, for the ethanolic extract of C. americana stems, moderate cytotoxicity was observed for Vero cells (CC_50_ = 54.4 μg/mL) and no cytotoxicity was observed for MRC-5 cells (CC_50_ > 400.0 μg/mL). Meanwhile, the leaf extract was considered noncytotoxic for both cell lines: Vero (CC_50_ = 161.5 μg/mL) and MRC-5 (CC_50_ > 400.0 μg/mL).

These cell lines are two nontumor transformed cell lines, and they have distinct characteristics. The Vero cell was deleted by -9-Mb on chromosome 12, which contained the CDKN2A and CDKN2B genes. CDKN2A encodes two cyclin-dependent kinase inhibitors, p16INK4A (which inhibits CDK6, a negative regulator of pRB retinoblastoma protein) and p14 ARF (which inhibits p53-negative regulator MDM2); CDKN2B already encodes the p15INK4B inhibitor (which inhibits another pKR CDK4 negative regulator). Loss of both genes plays a crucial role in the acquisition of immortality in the Vero cell line [38,39,40]. For MRC-5 cells, their immortality is related to the ectopic expression of the telomerase catalytic subunit (hTERT); the expression of this gene allows normal human cells to bypass senescence and, consequently, become immortal [41,42]. These differences may be related to the observed toxicity profile, which may indicate selective cytotoxic activity, an important parameter when searching for a substance with antineoplastic activity.

The evaluation of cytotoxicity in Vero cells is essential to be carried out prior to the antiviral activity assays to determine the concentration of extracts, in which cell viability corresponds to 50% (CC_50_), so that the antiviral assay can be performed from lower concentrations to CC_50_ [43].

MRC-5 cells are widely used in the assessment of cytotoxicity in parallel with biological activity screening assays due to their sensitivity and receptivity to various microorganisms [37]. Thus, the cytotoxicity in MRC-5 cells was performed with the purpose of inferring from this normal human cell line the cytotoxic potential of the extracts in human cells, and the low cytotoxicity in human cells is considered a favorable factor to proceed with the research of the antiviral activity of these extracts.

The results found in Vero cells for ethanolic extracts of *C. americana* stems and leaves in the present study are in line with literature data. De Toledo et al. [30] performed a Vero cell cytotoxicity test for the ethanolic extract of *C. americana* stems, and the result was very similar to that described in this study, with a CC_50_ of 53.3 μg/mL. In this work, the authors suggest that a moderate cytotoxic activity of this species is related to saponins and tannins present in the extracts [30]. According to Costa et al. [28], saponins interact strongly with the cell membrane, while tannins interact strongly with proteins, and these interactions are directly related to their chemical structures.

In the fractions obtained from the partition of the ethanolic extract of *C. americana* leaves, we can observe that the cytotoxicity results in Vero cells compared with MRC-5 cells are very different from each other. The EtOAc and DCM fractions showed moderately cytotoxic results for Vero cells with a CC_50_ of 56.2 and 58.9 μg/mL, respectively, while H_2_O/MeOH presented a CC_50_ of 101.0 μg/mL, which is considered noncytotoxic. For MRC-5 cells, no cytotoxicity was observed to the EtOAc and H_2_O/MeOH fractions (CC_50_ > 400.0 μg/mL) and to the DCM fraction (CC_50_ = 115.2 μ/mL). Despite that, according to Cos et al., the leaf extract fractions are not considered cytotoxic [37].

This cytotoxic difference can be explained by the fact that the transformations/mutations that occurred for each cell type with the goal of immortality are distinct from each other, directly impacting the selectivity of cytotoxicity, as occurs in stem and leaf extracts of this species [38,41]. Moreover, it is noteworthy that they are cell lines belonging to different animal species, which perform different functions in the organisms in which they live.

As for the isolated substances, quercetin-3-*O*-glucoside was not cytotoxic in the highest concentration tested (100.0 μg/mL) for both cell lines. Quercetin showed a CC_50_ of 235.8 μM (71.3 μg/mL) in Vero cell, and quercetin-3-*O*-hexosylgallate presented a CC_50_ of 496.1 μM (305.6 μg/mL) in Vero cell; also, it was noncytotoxic in the highest concentration tested (100.0 μg/mL).

These results of low or noncytotoxic activity are a favorable prognosis for testing biological activities, including antiviral, considering that when given a positive result, it can be said that the compound shows selectivity for infected cells.

After the determination of optimal noncytotoxic use concentrations, assays were performed, treating ZIKV-infected Vero cells with eight concentrations from the noncytotoxic concentrations for each sample. The results of the 50% effective concentration (EC_50_—concentration of the substance capable of reducing the viral cytopathic effect by 50%) and selectivity index (SI) are described in Table 2.

**Figure 3:**
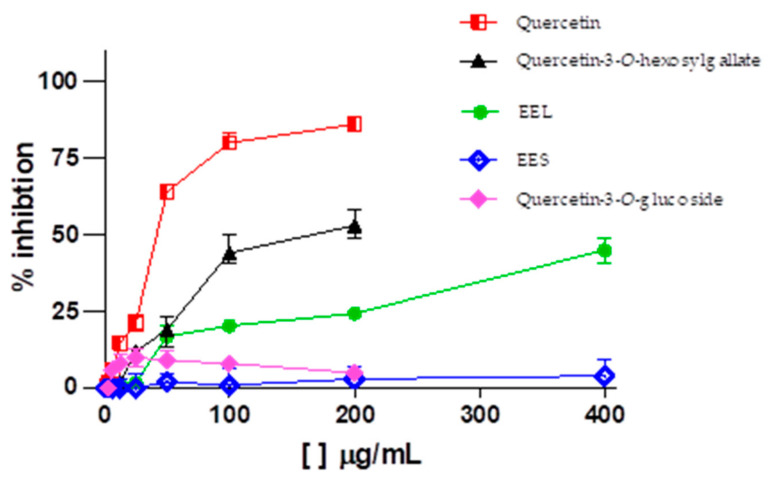
Dose–response curves for anti-Zika activity of ethanolic extracts and constituents from *Curatella americana* (EEL—ethanol extract of leaves, EES—ethanol extract of stems).

According to the chromatographic profile of *C. americana* leaf extract, we can observe that flavonoids-*O*-glycosylated were identified for this species. In general, flavonoids exhibit a multitude of biological activities, including antiviral activity against influenza viruses [44], hepatitis C [45], cytomegalovirus [46], dengue [47], Zika [48], and chikungunya [49]. Literature data show that flavonol-*O*-glycosylated, more specifically quercetin-3-*O*-glucoside, exhibits potent anti–Zika virus activity in Vero cell lines, A549 (human pulmonary epithelial cell), Huh-7 (human hepatoma cell), and SH-SY5Y (human neuroblastoma cell) [48,50]. In addition, it has recently been reported that epigallocatechin gallate (EGCG) and curcumin are also effective against ZIKV [49,51]. Thus, the phytochemical investigation sought the isolation of these flavonoids in the extract of leaves of *C. americana*, because they are believed to be responsible for the antiviral activity observed in the extract (Table 2 and Figure 3).

Corroborating with expectations, the ethyl acetate fraction, flavonoid rich, was the only fraction that showed significant anti–Zika virus activity with an EC_50_ of 40.7 µg/mL. There were three isolated substances obtained, where quercetin-3-*O*-hexosylgallate and quercetin registered activity against the Zika virus with an EC_50_ of 248.1 µM (152.8 µg/mL) and 61.6 µM (18.6 µg/mL), respectively, and quercetin-3-*O*-glucoside was not active (Figure 3).

Contrary to literature results, quercetin-3-*O*-glucoside isolated in this study did not show activity against Zika virus at the highest concentration tested (100.0 µg/mL) in the Vero cell line. This can be explained by the low quercetin-3-*O*-glucoside concentration used in the tests and increased viral titer compared with the study by Wong et at. [48], which used a concentration of 400.0 µg/mL quercetin-3-*O*-glucoside and an infection multiplicity of 0.05.

The activity of quercetin-3-*O*-hexosylgallate is believed to be related to the presence of the gallate group, given that this group was responsible for the anti–Zika virus activity determined for the flavonoid EGCG according to Sharma et al. [51].

Quercetin is a flavonol known for several properties, such as its antioxidant, antimicrobial, anticancer, antiviral, and anti-inflammatory effects. Recent studies have highlighted the potential use of quercetin as an antiviral, justified by its ability to inhibit the initial stages of virus infection, interact with proteases important for viral replication, and reduce inflammation caused by viral infection [52].

Our results corroborate with the literature, as demonstrated by Roy et al. [53] and Lim et al. [54], that quercetin is able to inhibit the ZIKV protease NS2B–NS3pro, which is absolutely essential for cleaving the ZIKV polyprotein into functional subunits. Additionally, the structure–activity relationship study performed by Zou et al. [55] demonstrated quercetin inhibiting the ZIKV infection by disrupting the virus entry and also by targeting the viral protease NS2B–NS3.

**Figure 4:**
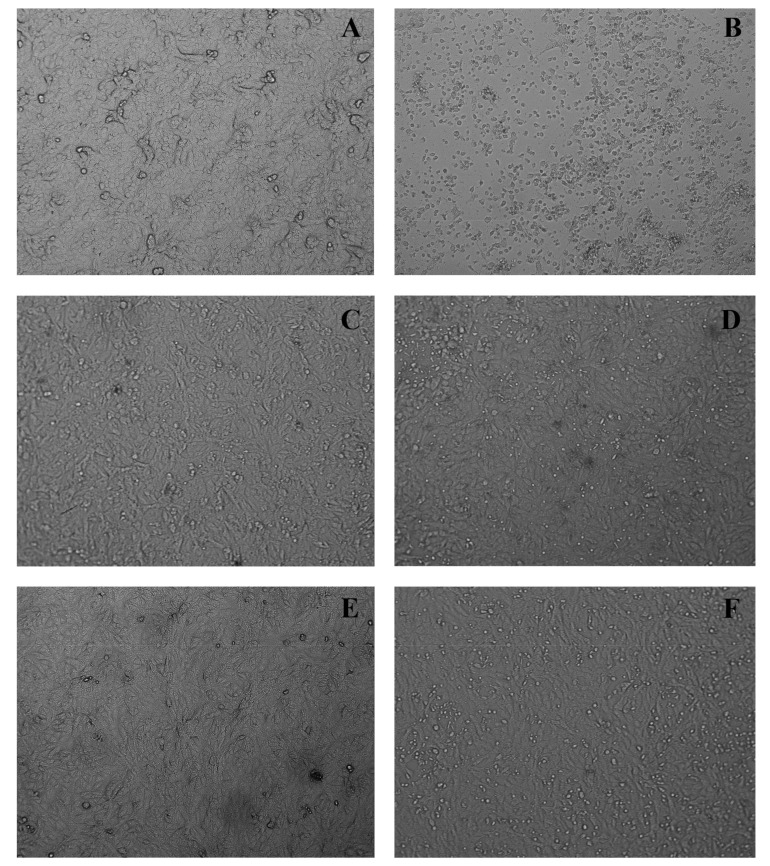
Antiviral effect against Zika virus in Vero cells treated with ethanol extract of leaves and quercetin. Vero cells were infected with ZIKV, treated with ethanol extract of leaves/quercetin, and photographed after 48 h of infection. (**A)** Uninfected and untreated cells, (**B**) infected cells, (**C**) cells uninfected and treated with ethanol extract of leaves (85 µg/mL), (**D**) cells infected and treated with ethanol extract of leaves (85 µg/mL), (**E**) cells uninfected and treated with quercetin (20 µg/mL), (**F**) cells infected and treated with quercetin (20 µg/mL). Magnification, 100x.

The results obtained in the in vitro cytopathic effect inhibition assay demonstrated that the cell monolayer infected by Zika virus allows viral multiplication inside the cells, leading to the cell monolayer destruction that was observed in the image (Figure 4B). This can be seen by the destruction and morphological alterations of the cells, such as rounding of cells, formation of lumps, and changes in cell refringence, especially when compared with the uninfected and untreated cell monolayer (Figure 4A).

While treating the ZIKV-infected cell monolayer with extracts and/or isolated quercetin, it is possible to protect the cells from a virus infection and thus prevent cell death once the cells remain attached (such as a monolayer) and no morphocellular deformities are observed. Therefore, they indicate that the treatment performed after infection is effective in inhibiting the virus multiplication cycle, confirming the antiviral activity observed in the MTT colorimetric assay.

This suggests that quercetin-like flavonoids are interesting drugs with selective and relevant in vitro anti–Zika virus activity. Therefore, future studies of these compounds may provide relevant data for the production of antivirus molecules.

## 3. Materials and Methods

### 3.1. General Experimental Procedures

The isolated compounds were submitted to ^1^D and ^2^D ^1^H and ^13^C-NMR spectra, such as COSY, HSQC, and HMBC, which were obtained on a Bruker Avance DRX 400 instrument (Bruker^®^, Billerica, MA, USA). Tetramethylsilane (TMS) was used as an internal reference, and deuterated methanol (CD_3_OD, Cambridge Isotope Laboratories, Inc.,^®^ Tewksbury, MA, USA) was used as a solvent.

In the TLC analyses on silica gel 60 (Merck^®^, Kenilworth, NJ, USA), ethyl acetate/formic acid/acetic acid/water (100:11:11:27) was used as mobile phase NP/PEG developer (Sigma-Aldrich^®^, San Luis, MO, USA) and as reference compounds quercetin (Sigma-Aldrich^®^, San Luis, MO, USA) and isoquercitrin (Sigma-Aldrich^®^, San Luis, MO, USA).

In preparing the samples for analysis by LC–MS, 2.5 mg of each extract and/or fractions were weighed directly into Eppendorf-type flasks, and 1.0 mL of ultrapure methanol (Merck^®^, Kenilworth, NJ, USA) was added. For the complete dissolution of the samples, sonication was used in an ultrasound bath; then the samples were centrifuged at 10,000 rpm for 5 min. The supernatant was filtered through 0.22 µm pore PVDF (polyvinylidene fluoride) filters and transferred to vials for use in CLUE-FR analysis.

### 3.2. Plant Material

The different parts of the plant (stems and leaves) of *Curatella americana* L. were collected in Santana de Pirapama (19°00′21′′ S and 44°02′34′′ W), Minas Gerais, Brazil. The taxonomic determinations were made by Prof. Maria Cristina Teixeira Braga Messias from the Department of Biodiversity, Evolution, and Environment in the Institute of Exact and Biological Sciences at the Federal University of Ouro Preto. The exsiccate was deposited at the Professor José Badini Herbarium (registration OUPR-UFOP 28723).

### 3.3. Preparation of Crude Extract and Fractionation

The different parts of the plants (leaves (1.2 kg) and stems (79.6 g)) were separated and dried in a ventilated oven at 40 °C. After drying, grinding was performed using a knife mill. The powders obtained were placed in amber glass bottles, each bottle being identified according to the part of the plant. The plant materials were subjected to exhaustive extraction by cold percolation with ethanol, obtaining extracts of stems (9.3 g) and leaves (490.0 g). The ethanolic extracts obtained were concentrated in a rotary evaporator (Buchi^®^, New Castle, DE, USA) under reduced pressure at a temperature of 45 °C. For complete removal of the solvent, the extract residues were transferred to previously weighed flasks and then carefully dried in an oven at 50 °C. The dry ethanolic extracts were submitted to cytotoxic and antiviral activity assays. Only the leaf extract inhibited the viral multiplication cycle, which was selected for the bioguided study.

**Figure 5:**
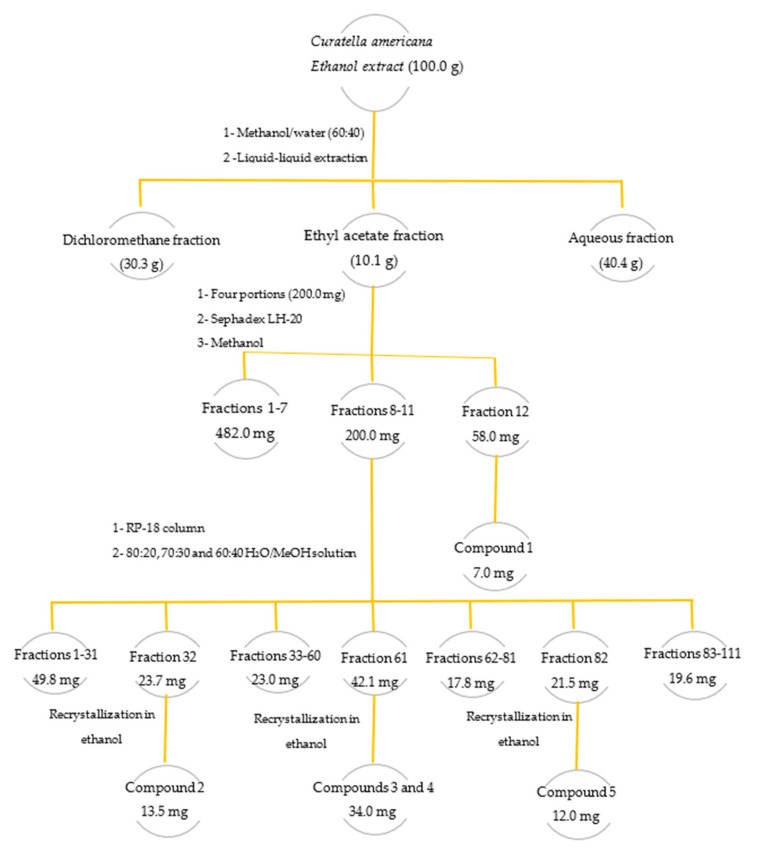
Fractionation of the ethanolic extract of leaves of the species *Curatella americana*.

In the preliminary fractionation of the leaf extract, the liquid–liquid partition technique with immiscible solvents was used. A portion of the extract (100.0 g) was solubilized in a methanol/water mixture (60:40) and sequentially partitioned with the organic solvents dichloromethane (DCM) and ethyl acetate (EtOAc) (3 times 100.0 mL each solvent). Thus, the DCM, EtOAc, and hydromethanolic (H_2_O/MeOH) fractions were obtained (Figure 5). These organic solutions were concentrated by distillation under reduced pressure on a rotary evaporator. The concentrated fractions obtained were transferred to previously weighed glass vials, dried in a 50 °C controlled temperature oven, and subsequently weighed.

### 3.4. Isolation and Identification of Flavonoids

Four portions of the ethyl acetate fraction (200.0 mg each) that was active in the antiviral assays were fractionated by size exclusion chromatography using Sephadex LH-20 resin (Pharmacia Biotech^®^, Raleigh, NC, USA) (Sephadex LH-20, 30.0 g, ø 70.0 μm; eluted with MeOH). In the fractionation of each portion, 40 fractions of 10.0 mL on average were collected. The fractions were subjected to analysis by thin-layer chromatography (TLC), and those with similar profiles were pooled. From the pooled fraction number 12, a yellow solid identified as quercetin-3-*O*-hexosylgallate (**1**) (7.0 mg) was obtained (Figure 5).

Fractions 8 to 11 with a similar profile on TLC were pooled (totaling 200.0 mg) and subjected to fractionation by chromatography on a reversed-phase silica gel octadecylsilane column (Merck^®^, Kenilworth, NJ, USA, RP-18 column, 4 g, ø 10 µm; eluted with an 80:20, 70:30 and 60:40 H_2_O/MeOH solution). A total of 220 fractions of approximately 5.0 mL were collected, grouped into 111 fractions after analysis by TLC. Fraction 32 resulted in the isolation of a yellow solid, called quercetin-3-*O*-glucoside (**2**), known as isoquercitrin (13.5 mg). In pooled fraction 61, a 34.0 mg mixture of the flavonoids quercetin-3-*O*-rhamnoside (**3**) and quercetin-3-*O*-arabinoside (**4**) were obtained. Fraction 82 was recrystallized with absolute ethanol from which 12.0 mg of a yellow solid identified as quercetin (**5**) was obtained (Figure 5).

The isolated compounds were identified by nuclear magnetic resonance (^1^H NMR and ^13^C NMR). The data were confirmed by two-dimensional techniques (DEPT, COSY, HSQC, HMBC) and by mass spectrometry. Additionally, the data obtained in this study were compared with data from the literature [56,57,58,59].

### 3.5. Spectroscopic Data for Isolated Compounds

Quercetin-3-*O*-hexosylgallate (compound 1): Yellowish powder, UV λ_max_ 264, 354 nm; ESI-MS *m*/*z*: [M-H]^+^ 617.22, HRMS [M+H]^+^ 617.1137, cald. for C_28_H_25_O_16_ 617.1142 Da; ^1^H-NMR (CD_3_OD, 400 MHz, ppm) 6.18 (1H, d, *J* = 2.24 Hz, H-6), 6.37 (1H, d, *J* = 1.96 Hz, H-8), 7.78 (1H, d, *J* = 2.12 Hz, H-2′), 6.81 (1H, d, *J* = 8.48 Hz, H-5′), 7.55 (1H, m, H-6′), 5.11 (1H, d, *J* = 7.8 Hz, H-1′′), 3.85 (1H, m, H-2′′), 3.80 (1H, m, H-3′′), 3.87 (1H, m, H-4′′), 3.59 (1H, m, H-5′′), 4.09 (2H, q, H-6′′), 6.88 (2H, s, H-2′′′, H-6′′′); ^13^C-NMR (CD_3_OD, 400 MHz, ppm) 158.3 (C-2), 135.7 (C-3), 179.5 (C-4), 166.0 (C-5), 99.9 (C-6), 168.0 (C-7), 94.8 (C-8), 162.8 (C-9), 105.4 (C-10), 123.6 (C-1′), 117.7 (C-2′), 145.8 (C-3′), 149.9 (C-4′), 116.1 (C-5′), 123.0 (C-6′), 105.5 (C-1′′), 73.0 (C-2′′), 74.5 (C-3′′), 70.0 (C-4′′), 74.9 (C-5′′), 61.6 (C-6′′), 121.0 (C-1′′′), 109.8 (C-2′′′, 6′′′), 146.3 (C-3′′′), 145.8 (C-5′′′), 135.7 (C-4′′′), 173.1 (C-7′′′).

Quercetin-3-*O*-glucoside (compound 2): Yellowish powder, UV λ_max_ 255, 354 nm; ESI-MS m/z: [M+H]^+^ 465.13, HRMS [M+H]^+^ 465.1030, cald. for C_21_H_21_O_12_ 465.1033; 1H NMR (400 MHz, CD_3_OD, ppm) 6.26 (1H, d, *J* = 2,16 Hz, H-6), 6.08 (1H, d, *J* = 2.08 Hz, H-8), 7.59 (1H, d, *J* = 2.16 Hz, H-2′), 6.74 (1H, d, *J* = 8.48 Hz, H-5′), 7.46 (1H, dd, *J* = 8.48, 2.16 Hz, H-6′), 5.14 (1H, d, *J* = 7.4 Hz, H-1′′), 3.36 (1H, m, H-2′′), 3.34 (1H, m, H-3′′), 3.31 (1H, m, H-4′′), 3.11 (1H, m, H-5′′), 3.58 (1H, dd, *J* = 11.92, 2.16 Hz, Ha-6′′), 3.61 (1H, dd, *J* = 11.88, 5.2 Hz, Hb-6′′); ^13^C NMR (400 MHz, CD_3_OD, ppm) 158.6 (C-2), 135.7 (C-3), 179.6 (C-4), 163.1 (C-5), 100.0 (C-6), 166.1 (C-7), 94.8 (C-8), 159.1 (C-9), 105.8 (C-10), 123.3 (C1′), 117.7 (C-2′), 146.0 (C-3′), 149.9 (C-4′), 116.1 (C-5′), 123.2 (C-6′), 104.4 (C-1′′), 75.8 (C-2′′), 78.2 (C-3′′), 71.3 (C-4′′), 78.5 (C-5′′), 62.6 (C-6′′).

Quercetin-3-*O*-rhamnoside (compound 3): Yellowish powder, UV λ_max_ 256 and 349 nm; ESI–MS m/z: [M+H]^+^ 449.13, HRMS [M+H]^+^ 449.1080, cald. for C_21_H_21_O_11_ 449.1083 Da; ^1^H NMR (400 MHz, CD_3_OD, ppm) 6.09 (1H, m, H-6), 6.27 (1H, m, H-8), 6.82 (1H, m, H-2′), 7.24 (1H, m, H-5′), 7.22 (1H, m, H-6′), 5.25 (1H, m, H-1′′), 3.80 (1H, m, H-2′′), 4.12 (1H, m, H-3′′), 3.32 (1H, m, H-4′′), 3.66 (1H, m, H-5′′), 0.83 (1H, m, H-6′′); ^13^C NMR (100 MHz, CD_3_OD, ppm), 159.4 (C-2), 136.4 (C-3), 179.6 (C-4), 163.2 (C-5), 100.0 (C-6), 166.2 (C-7), 94.8 (C-8), 158.5 (C-9), 105.7 (C-10), 123.1 (C1′), 116.5 (C-2′), 146.6 (C-3′), 149.9 (C-4′), 117.0 (C-5′), 123.0 (C-6′), 103.7 (C-1′′), 73.0 (C-2′′), 72.0 (C-3′′), 72.2 (C-4′′), 72.1 (C-5′′), 17.8 (C-6′′).

Quercetin-3-*O*-arabinoside (compound 4): Yellowish powder, UV λ_max_ 256 and 354 nm; ESI–MS m/z: [M+H]+ 435.15, HRMS [M+H]^+^ 435.0922, cald. for C_20_H_19_O_11_ 435.0927 Da; ^1^H NMR (400 MHz, CD_3_OD, ppm) 6.10 (1H, m, H-6), 6.29 (1H, m, H-8), 6.79 (1H, m, H-2′), 7.65 (1H, m Hz, H-5′), 7.48 (1H, m, H-6′), 5.05 (1H, m, H-1′′), 3.25 (1H, m, H-2′′), 3.55 (1H, m, H-3′′), 3.71 (1H, m, H-4′′), 3.72/3.35 (2H, m, H-5′′); ^13^C NMR (100 MHz, CD_3_OD, ppm), 158.8 (C-2), 135.8 (C-3), 179.8 (C-4), 163.3 (C-5), 99.9 (C-6), 166.3 (C-7), 94.8 (C-8), 158.7 (C-9), 106.0 (C-10), 123.1 (C1′), 116.3 (C-2′), 146.1 (C-3′), 150.1(C-4′), 117.6 (C-5′), 123.1 (C-6′), 104.8 (C-1′′), 73.4 (C-2′′), 74.3 (C-3′′), 69.3 (C-4′′), 67.1 (C-5′′).

Quercetin (compound 5): Yellowish powder, UV λ_max_ 274 and 369 nm; ESI–MS m/z: [M+H]^+^ 303.06, HRMS [M+H]^+^ 303.0498, cald. for C_15_H_11_O_7_ 303.0504 Da; ^1^H NMR (400 MHz, CD_3_OD, ppm) 6.01 (1H, d, *J* = 2.08 Hz, H-6), 6.21 (1H, d, *J* = 1.96 Hz, H-8), 7.56 (1H, d, *J* = 2.12 Hz, H-2′), 6.71 (1H, d, *J* = 8.48 Hz, H-5′), 7.47 (1H, dd, *J* = 7.44, 2.16 Hz, H-6′); ^13^C NMR (100 MHz, CD_3_OD, ppm), 148.9 (C-2), 137.4 (C-3), 177.4 (C-4), 162.6 (C-5), 99.4 (C-6), 165.7 (C-7), 94.5 (C-8), 158.3 (C-9), 104.6 (C-10), 124.3 (C1′), 116.1 (C-2′), 146.3 (C-3′), 148.1(C-4′), 116.4 (C-5′), 121.8 (C-6′).

### 3.6. LC–DAD–MS and LC–ESI–MS/MS Analyses

Liquid chromatography analyses were performed on a UPLC Acquity^®^ (Waters, Milford, MA, USA) ion trap mass spectrometer according to the method developed by Fonseca et al. [60]. The analysis conditions were as follows: positive and negative ion mode; capillary voltage, 3500 V; capillary temperature, 320 °C; source voltage, 5 kV; vaporizer temperature, 320 °C; corona needle current, 5 mA; and sheath gas, nitrogen, and 27 psi. The analyses were run in full scan mode (100–2000 Da). The ESI–MS/MS analyses were additionally performed in a UPLC Acquity (Waters, Milford, MA, USA) with argon as the collision gas, and the collision energy was set at 30 eV. Chromatographic separation was performed on an Acquity UPLC BEH (1.7 μm, 50 × 2 mm i.d.) (Waters, Milford, MA, USA). The mobile phase consisted of water, 0.1% formic acid (solvent A), and acetonitrile 0.1% formic acid (solvent B). The elution protocol was 0–11 min, the linear gradient from 5% to 95% B. The flow rate was 0.3 mL min−1, and the sample injection volume was 4.0 μL. The UV spectra were registered from 190 to 450 nm. Mass spectrometry analysis was performed utilizing a Waters Acquity^®^ TQD equipped (Waters, Milford, MA, USA) with a quadrupole instrument fitted in an electrospray source on a positive and negative ESI mode. Ion spray voltage: −4 kV; orifice voltage: −60 V.

### 3.7. High-Resolution Mass Spectrometry Analyses

High-resolution mass spectrometry analyses were performed according to the method described by Cruz et al. [21]. A Nexera UHPLC system (Shimadzu, Kyoto, Japan) combined with a maXis high-resolution ESI-QTOF mass spectrometer (Bruker) controlled by the Compass 1.7 software package (Bruker). A 5 μg sample was injected into a Shimadzu Shim-pack XR-ODS III column (C18, 2.2 µm, 2.0 × 150 mm) at 40 °C, with a flow rate of 400 μL/min. Mobile phases A and B (0.1% formic acid in Milli-Q water and acetonitrile, respectively) formed an eluent gradient from an initial 5 min of 5% B to 100% B in 40 min, with a hold at 100% B for 5 min. After UV–PDA detection (190–450 nm), mass spectra were acquired in positive mode at a rate of 5 Hz. Ion-source parameters were set to 500 V end-plate offset, 4500 V capillary voltage, 3.0 bar nebulizer pressure, and 8 L/min and 200 °C dry gas flow and temperature, respectively. Data-dependent fragment spectra were recorded using a collision energy range between 15 and 60 eV. Ion cooler settings were optimized for an *m*/*z* 100–1500 range, using a calibrant solution of 1 mM sodium formate in 50% 2-propanol. Mass calibration was achieved by initial ion-source infusion of 20 μL calibrant solution and postacquisition recalibration of the raw data. Compound detection was achieved by chromatographic peak dissection with subsequent formula determination according to the exact mass and isotope pattern (MS1). Putative identification was based on comparison of compound fragment spectra (MS2) with reference spectra from an in-house database of standard compounds (Fiocruz Minas), the public spectrum database MassBank, and in silico fragment spectra generated from the Universal Natural Product database [61,62].

### 3.8. Cytotoxicity Evaluation by MTT Assay

Cell suspensions (Vero and MRC-5) were distributed in 96-well microplates containing 6.0 × 10^4^ cells/well. The plates were incubated in a humidified 5% CO_2_ atmosphere at 37 °C for 24 h. Samples solubilized in dimethyl sulfoxide (DMSO) were diluted in culture medium supplemented with 1% SFB [48,49]. Substance dilutions ranged from 800 to 0.125 µg/mL. After the formation of the cell monolayer on the surface of the wells, the culture medium was removed, and 100 μL of diluted sample solutions along with 100 μL of 1% SFB-enriched culture medium were added, and the plates were incubated under the same atmospheric condition [63,64]. Seventy-two hours after the addition of samples, the culture medium was removed, and 28.0 μL of MTT solution (2.0 mg/mL in PBS) was added to each well, and the plates were incubated again for 120 min. At the end of this period, 132 μL of DMSO was added to all wells, and the plates were shaken on a plate shaker for 15 min to dissolve the formed formazan [63,64]. Formazan quantification obtained by reducing tetrazolium salt in viable cells was performed on a Victor^TM^ X3 microplate reader^®^ (PerkinElmer, Waltham, MA, USA) using the WorkOut 2.5 software at 490 nm. Cell multiplication was compared with cell control. Cell toxicity was expressed in terms of 50% cytotoxic concentration (CC_50_). The cytotoxic percentage was calculated as [(A-B)/A] × 100, where A and B are the optical densities at 490 nm (OD_490_) from the wells where untreated (A) and treated (B) cells are present, respectively.

### 3.9. Evaluation of Anti–Zika Virus Activity

#### 3.9.1. Preparation of Viral Suspensions

Zika virus (ZIKV) was picked on C6/36 cells to generate working stocks for 5 days at 28 °C. The infected cell culture supernatant was harvested after 5 days and clarified by centrifugation at 4000 RPM and refrigerated for 10 min at 4 °C, and then aliquoted and stored at −70 °C as virus stock until use.

#### 3.9.2. Determination of Viral Infectious Titer by TCID_50_

After the incubation period (24 h), for the formation of the Vero cell monolayer, the viral suspension was infected, containing in each well 100.0 μL of each dilution in 1% SFB DMEM medium. Viral stocks were diluted (10-1 to 10-18) and inoculated into Vero cell monolayers implanted in approximately 85% confluence 96-well plates (6 × 10^4^ cells per well), maintaining three uninfected columns for cell control. The plates were then incubated at 37 °C (5% CO_2_), and the cytopathic effect (ECP) was monitored. ECP was recorded every 24 h for 72 h, observing cell control.

#### 3.9.3. Screening for Antiviral Activity by MTT Colorimetric Technique

It was performed according to the methodology described by Brandão et al. [30]. Sample stock solutions were prepared in DMSO (dimethyl sulfoxide) and evaluated at noncytotoxic concentrations. Vero cell monolayers were cultured in 96-well plates (6 × 10^4^ cells per well). After 24 h of incubation in 5% CO_2_ at 37 °C, the culture medium (DMEM 5% SFB) was removed, and 100.0 μL of sample dilutions were added to each well along with 100.0 μL of the viral suspension. Then, the cultures were incubated in a humid atmosphere containing 5% CO_2_ at 37 °C for 72 h. Cell viability was assessed by the MTT colorimetric technique [37]. The supernatant was removed, and then 28.0 μL of an MTT solution (2.0 mg/mL in PBS) was added to each well. The plates were incubated for 2 h at 37 °C, and after this incubation time, 132.0 μL DMSO was added to each well to dissolve the formazan crystals. The plates were stirred for 15 min (Shaker New Brunswick Scientific C24), and the optical density was determined at 490 nm (OD_490_) in a microplate reader.

The effective concentration that has 50% antiviral effect, or 50% effective concentration (EC_50_), is expressed as the concentration that promotes the protection of 50% of treated cells from destruction caused by the virus. The percentage of protection was calculated as [(AB)/CB)] × 100, where A, B, and C are the OD_490_ of the wells, in which are present treated and infected (A), untreated and infected (B), and untreated and non-infected cells (C), respectively.

The selectivity index (SI), corresponding to the therapeutic index, is defined as the ratio of CC_50_ to EC_50_ values, and it shows the selectivity of the samples in inhibiting the virus multiplication cycle [37].

#### 3.9.4. In Vitro Cytopathic Effect Inhibition Assay

In order to confirm the anti-ZIKV activity observed in the antiviral MTT assay, the Vero cell monolayer (9.5 × 10^6^ cells per well) was infected by viral suspensions with titers of 1.0 × 10^7^ TCID_50_/mL, (MOI = 1), Zika virus, during 1 h for the viral adsorption. Afterwards, the viral suspension was removed and the wells were washed with PBS. Then, the wells were treated with the active concentration of the compound (20 μg/mL) and ethanolic extract of leaves of *C. americana* (85 μg/mL). The plates were incubated at 37 °C in a humidified 5% CO_2_ atmosphere and photographed 48 h after infection [17].

### 3.10. Statistical Analysis

Data from cytotoxicity and antiviral assays were evaluated according to their means and standard deviations. The cytotoxic concentration at 50% and the effective concentration at 50% were determined compared with the control obtained from nonlinear regression. These analyses were performed using the GraphPad Prism 5.04 statistical package^®^ (GraphPad Software, Boston, MA, USA).

## 4. Conclusions

*Curatella americana* leaves are rich in phenolic compounds, mainly flavonoids. The bioguided phytochemical investigation of the ethanolic extract of *C. americana* leaves revealed that medium polarity constituents may be responsible for the observed anti-ZIKV activity, considering that only the ethyl acetate fraction obtained from the liquid–liquid partition of the ethanolic extract was active against ZIKV. From this fraction, three flavonoids were isolated: quercetin-3-*O*-hexosylgallate, quercetin-3-*O*-glucoside, and quercetin, in addition to a mixture (quercetin-3-*O*-rhamnoside and quercetin-3-*O*-arabinoside). Flavonoids can be considered primarily responsible for the antiviral activity observed, since the active substances obtained, quercetin-3-*O*-hexosylgallate and quercetin, showed significant biological activity, exhibiting selective and promising anti-ZIKV activity in vitro.

## Figures and Tables

**Table 1:** Spectroscopic data and characterization of polyphenols in leaf ethanolic extract from *Curatella americana* by UPLC–ESI–MS/MS.

	Compounds	Molecular Formula	RT * (min)	UV (nm)	[M-H]^−^ (*m*/*z*)	Characteristic *m*/*z* of Ions in Positive Ion Mode (%)	HRMS [M + H]^+^ (*m*/*z*)	Error (ppm)
1	(Epi)catechin	C_15_H_14_O_6_	13.1	278	288.95	291.0860 (30.02), 139.0387 (100.0), 123.0437 (45.4)	291.0858	3.4
2	(Epi)catechin	C_15_H_14_O_6_	14.3	279	289.14	291.0859 (34.8), 147.0439 (24.3), 139.0386 (100.0)	291.0859	3.1
3	B-type dimer of (epi)catechin-(epi)catechin-*O*-gallate	C_37_H_30_O_16_	14.4	277	729.27	731.1636 (16.4), 579.1163 (13.4), 291.0860 (12.8), 153.0180 (100.0)	731.1607	0.7
4	Homoorientin	C_21_H_20_O_11_	15.3	265, 350	447.17	449.1087 (73.1), 329.0650 (71.7), 299.0546 (100.0)	449.1074	2.0
5	Orientin	C_21_H_20_O_11_	15.5	265, 350	447.26	449.1084 (100.0), 329.0651 (67.0), 299.0546 (38.7)	449.1077	1.3
7	Quercetin-3-*O*-hexosyl-arabinoside	C_26_H_28_O_16_	15.5	269, 350	595.33	597.1469 (1.8), 465.1037 (7.7), 303.0500 (100.0)	597.1449	1.8
8	Quercetin-3-*O*-hexosylgallate	C_28_H_24_O_16_	16.0	264, 354	615.20	617.1156 (18.8), 303.0500 (97.3), 153.0182 (100.0)	617.1137	1.0
9	Isovitexin	C_21_H_20_O_10_	16.1	267, 350	431.09	433.1138 (100.0), 313.0708 (59.2), 283.0600 (42.9)	433.1129	1.1
10	Vitexin	C_21_H_20_O_10_	16.2	267, 351	431.23	433.1137 (100.0), 313.0709 (57.3), 283.0601 (42.2)	433.1130	0.9
11	Quercetin-3-*O*-glucoside	C_21_H_20_O_12_	16.3	256, 354	463.17	465.1035 (2.0), 303.0501 (100.0)	465.1030	0.6
12	Kaempferol-3-*O*-hexosyl-arabinoside	C_26_H_28_O_15_	16.4	256, 354	579.20	449.1083 (11.1), 287.0552 (100.0)	581.1499	1.2
13	Kaempferol-3-*O*-hexosylgallate	C_28_H_24_O_15_	16.5	269,353	599.06	601.1212 (7.4), 287.0549 (100.0), 153.0181 (75.5)	601.1185	1.3
14	Quercetin-3-*O*-arabinoside	C_20_H_18_O_11_	16.8	256, 354	433.32	435.0927 (1.2), 303.0500 (100.0), 153.0182 (2.7)	435.0922	1.1
15	Methylquercetin-3-*O*-glucuronylgallate	C_29_H_26_O_16_	16.8	270, 365	629.24	631.1308 (9.5), 329.0868 (19.1), 303.0500 (59.7), 167.0341 (100.0)	631.1291	1.3
16	Kaempferol-3-*O*-hexosylgallate	C_28_H_24_O_15_	17.1	257, 354	599.25	601.1209 (19.7), 315.0711 (18.8), 287.0551 (99.3), 153.0183 (100.0)	601.1189	0.7
17	Kaempferol-3-*O*-glucoside	C_21_H_20_O_11_	17.4	256, 349	447.30	287.0551 (100.0), 153.0182 (4.4)	449.1079	0.9
18	Quercetin-3-*O*-rhamnoside	C_21_H_20_O_11_	17.5	256, 348	447.17	303.0503 (100.0), 153.0182 (11.7)	449.1080	0.7
19	Quercetin-3-*O*-hexosyl-*O*-acetyl	C_23_H_22_O_13_	17.6	256, 354	505.18	507.1143 (8.4), 303.0501 (100.0), 187.0599 (6.3)	507.1133	1.0
20	Kaempferol-3-*O*-arabinoside	C_20_H_18_O_10_	17.7	256, 354	417.31	419.0967 (2.1), 287.0551 (100.0), 153.0184 (3.7)	419.0973	1.2
21	Quercetin-3-*O*-hexosyl-*O*-acetyl	C_23_H_22_O_13_	18.0	256, 354	505.21	507.1145 (3.6), 303.0501 (100.0), 205.0707 (3.3)	507.1135	0.6
22	Kaempferol-3-*O*-rhamnoside	C_21_H_20_O_10_	18.5	270, 355	431.29	287.0552 (100.0), 153.0182 (2.6)	433.1130	0.9
23	Quercetin-3-*O*-arabinosyl-O-acetyl	C_22_H_20_O_12_	18.7	256, 354	475.13	303.0501 (100.0), 175.0602 (12.2)	477.1028	1.0
24	Kaempferol-3-*O*-hexosyl-*O*-acetyl	C_23_H_22_O_12_	18.8	256, 354	489.17	287.0550 (100.0), 187.0603 (5.9)	491.1184	1.0
25	Quercetin-3-*O*-(6”-*O*-*E*-*p*-coumaroyl)-glucopyranoside	C_30_H_26_O_14_	19.2	277, 354	609.25	611.1407 (4.9), 303.0500 (25.8), 147.0441 (100.0)	611.1394	2.3
26	Quercetin	C_15_H_10_O_7_	20.1	275, 371	301.29	303.0500 (100.0), 229.0496 (7.4), 153.0182 (10.5), 137.0232 (4.5)	303.0498	2.0
27	Kaempferol-3-O-(6”-*O*-*E*-*p*-coumaroyl)-glucopyranoside	C_30_H_26_O_13_	20.2	277, 350	593.18	595.1456 (4.2), 309.0968 (15.3), 287.0549 (29.4), 147.0441 (100.0)	595.1446	0.8
28	Kaempferol-3-O-(2”-*O*-*E*-*p*-coumaroyl)-glucopyranoside	C_30_H_26_O_13_	20.6	254, 369	593.31	595.1459 (7.6), 309.0965 (7.3) 287.0549 (65.4), 147.0440 (100.0)	595.1444	1.2
29	Kaempferol	C_15_H_10_O_6_	22.0	269, 350	285.09	287.0550 (100.0), 153.0180 (14.5), 137.0232 (2.2)	287.0548	2.4

* Retention time obtained in chromatographic/HRMS analysis.

**Table 2:** Anti–Zika virus activity and its standard deviations (*n* = 3) of ethanolic extracts of stems and leaves, fractions, substances isolated from *Curatella americana*.

Extracts	Used Part	CC_50_ Vero µg/mL	CC_50_ MRC-5 µg/mL	EC_50_ µg/mL	SI Vero	SI MRC-5
*Curatella americana*	Stems	54.6 ± 1.4	>400	Inactive	-	-
*Curatella americana*	Leaves	161.5 ± 2.0	>400	85.2 ± 1.6	1.9	>2.5
		**Fractions**				
Fraction DCM	Leaves	58.9 ± 1.3	115.2 ± 1.8	Inactive	-	-
Fraction AcOEt	Leaves	56.2 ± 1.2	>400	40.7 ± 2.3	1.4	9.8
Fraction H_2_O/MeOH	Leaves	101.0 ± 1.2	>400	Inactive	-	-
		**Isolated Compounds**				
Quercetin-3-*O*-hexosylgallate	Leaves	305.6 ± 1.4 (496.1 ± 1.4 µM)	>100 (>162.3 µM)	152.8 ± 2.0 (248.1 ± 2.0 µM)	2.0	>0.7
Quercetin-3-*O*-glucoside	Leaves	>100 (>215.5 µM)	>100 (>215.5 µM)	Inactive	-	-
Quercetin	Leaves	71.3 ± 1.3 (235.8 ±1.3 µM)	NT	18.61 ± 2.8 (61.6 ± 2.8 µM)	3.8	-
Ribavirin (positive control)	-	370.4 ± 1.2 (1516.7 ± 1.2 µM)	NT	94.47 ± 2.7 (386.8 ± 2.7 µM)	3.9	-

## Data Availability

Not applicable.

## Supplementary Materials

The following supporting information can be downloaded at: https://www.mdpi.com/article/10.3390/molecules28062546/s1. Figure S1: ^1^H NMR spectrum of Quercetin-3-*O*-hexosylgalate (**1**) (400 Hz, CD_3_OD, δ); Figure S2: ^13^C NMR spectrum of Quercetin-3-*O*-hexosylgalate (**1**) (100 Hz, CD_3_OD, δ); Figure S3: DEPT-135° NMR spectrum of Quercetin-3-*O*-hexosylgalate (**1**) (100 Hz, CD_3_OD, δ); Figure S4: COSY contour map of Quercetin-3-*O*-hexosylgalate (**1**) (400 Hz, CD_3_OD, δ); Figure S5: HSQC contour map of Quercetin-3-*O*-hexosylgalate (**1**) (400 Hz, CD_3_OD, δ); Figure S6: HMBC contour map of Quercetin-3-*O*-hexosylgalate (**1**) (400 Hz, CD_3_OD, δ); Figure S7 ^1^H NMR spectrum of Quercetin-3-*O*-glucoside (**2**) (400 Hz, CD^3^OD, δ); Figure S8: ^13^C NMR spectrum of Quercetin-3-*O*-glucoside (**2**) (100 Hz, CD_3_OD, δ); Figure S9: DEPT-135° NMR spectrum of Quercetin-3-*O*-glucoside (**2**) (100 Hz, CD_3_OD, δ); Figure S10: COSY contour map of Quercetin-3-*O*-glucoside (**2**) (400 Hz, CD_3_OD, δ); Figure S11: HSQC contour map of Quercetin-3-*O*-glucoside (**2**) (400 Hz, CD_3_OD, δ); Figure S12: HMBC contour map of Quercetin-3-*O*-glucoside (**2**) (400 Hz, CD_3_OD, δ); Figure S13: ^1^H NMR spectrum of Quercetin-3-*O*-rhamnoside (**3**) (400 Hz, CD_3_OD, δ); Figure S14: ^13^C NMR spectrum of Quercetin-3-*O*-rhamnoside (**3**) (100 Hz, CD_3_OD, δ); Figure S15: DEPT-135° NMR spectrum of Quercetin-3-*O*-rhamnoside (**3**) (100 Hz, CD_3_OD-d6, δ); Figure S16: COSY contour map of Quercetin-3-*O*-rhamnoside (**3**) (400 Hz, CD_3_OD, δ); Figure S17: HSQC contour map of Quercetin-3-*O*-rhamnoside (**3**) (400 Hz, CD_3_OD, δ); Figure S18: HMBC contour map of Quercetin-3-*O*-rhamnoside (**3**) (400 Hz, CD_3_OD, δ); Figure S19: ^1^H NMR spectrum of Quercetin-3-*O*-arabinoside (**4**) (400 Hz, CD_3_OD, δ); Figure S20: ^13^C NMR spectrum of Quercetin-3-*O*-arabinoside (**4**) (100 Hz, CD_3_OD, δ); Figure S21: DEPT-135° NMR spectrum of Quercetin-3-O-arabinoside (**4**) (100 Hz, CD_3_OD-d6, δ); Figure S22: COSY contour map of Quercetin-3-O-arabinoside (**4**) (400 Hz, CD_3_OD-d6, δ); Figure S23: HSQC contour map of Quercetin-3-*O*-arabinoside (**4**) (400 Hz, CD_3_OD, δ); Figure S24: HMBC contour map of Quercetin-3-*O*-arabinoside (**4**) (400 Hz, CD_3_OD, δ); Figure S25: ^1^H NMR spectrum of Quercetin (**5**) (400 Hz, CD_3_OD, δ); Figure S26: ^13^C NMR spectrum of Quercetin (**5**) (100 Hz, CD_3_OD, δ); Figure S27: COSY contour map of Quercetin (**5**) (400 Hz, CD_3_OD, δ); Figure S28: HSQC contour map of Quercetin (**5**) (400 Hz, CD_3_OD, δ); Figure S29: HMBC contour map of Quercetin (**5**) (400 Hz, CD_3_OD, δ).
