# Supplementary material for: Anti–Zika Virus Activity and Isolation of Flavonoids from Ethanol Extracts of Curatella americana L. Leaves
Source: Molecules. 2023 Mar 10;28(6):2546. doi: 10.3390/molecules28062546 (PMC10054362; doi:10.3390/molecules28062546)
Supplement: Supplementary file 1 [file molecules-28-02546-s001.zip › molecules-2231916-supplementary.pdf]

molecules

MDPI

Supplementary Materials

Anti–Zika Virus Activity and Isolation of Flavonoids from Ethanol Extracts of *Curatella americana* L. Leaves

Lienne D'Auria Lima [1], Adriana Cotta Cardoso Reis [1], Jordano Augusto Carvalho Sousa [1], Gabriel Mendonça Valente [1], Breno de Mello Silva [2], Cíntia Lopes de Brito Magalhães [2], Markus Kohlhoff [3], Luiz Fernando de Medeiros Teixeira [4] and Geraldo Célio Brandão [1,*]

[1] Departamento de Farmácia, Escola de Farmácia, Universidade Federal de Ouro Preto, Campus Morro do Cruzeiro, Ouro Preto 35.400-000, MG, Brazil
[2] Departamento de Ciências Biológicas, Instituto de Ciências Exatas e Biológicas,, Universidade Federal de Ouro Preto, Campus Morro do Cruzeiro, Ouro Preto 35.400-000, MG, Brazil;
[3] Laboratório de Química de Produtos Naturais Bioativos, Fundação Oswaldo Cruz. Instituto René Rachou, Belo Horizonte 30.190-009, MG, Brazil
[4] Departamento de Análises Clínicas, Escola de Farmácia, Universidade Federal de Ouro Preto, Campus Morro do Cruzeiro, Ouro Preto 35.400-000, MG, Brazil

* Correspondence: celiobrandao@ufop.edu.br; Tel.: +55-31-3559-1088; Fax: +55-31-3559-

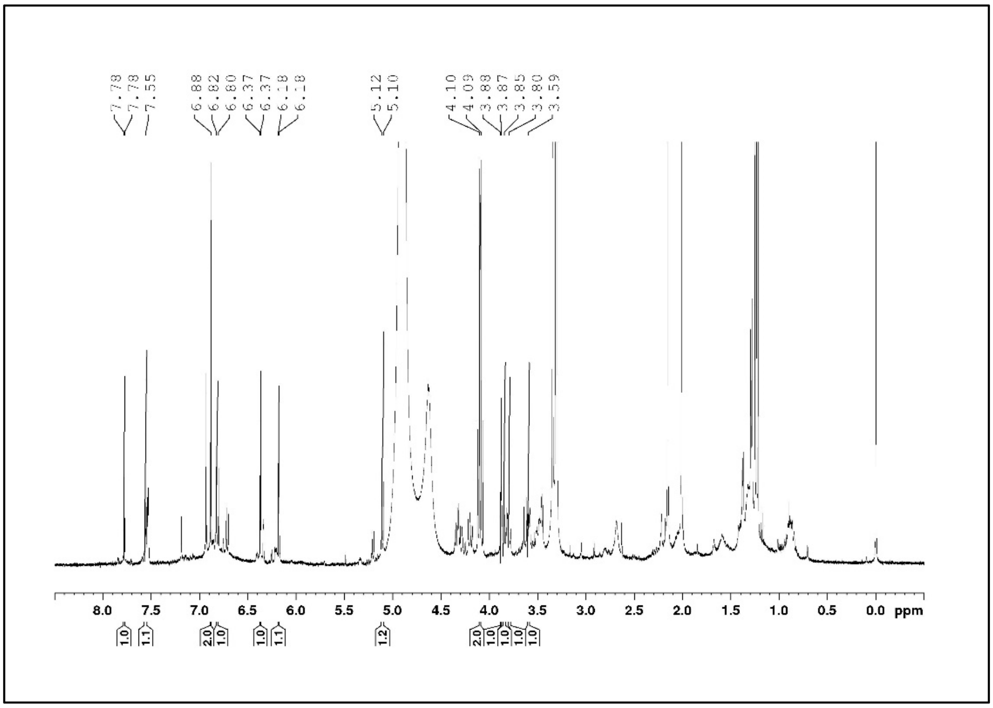

Figure S1. – ^{1}H NMR spectrum of Quercetin-3-*O*-hexosylgalate (**1**) (400 Hz, CD_3OD, δ).

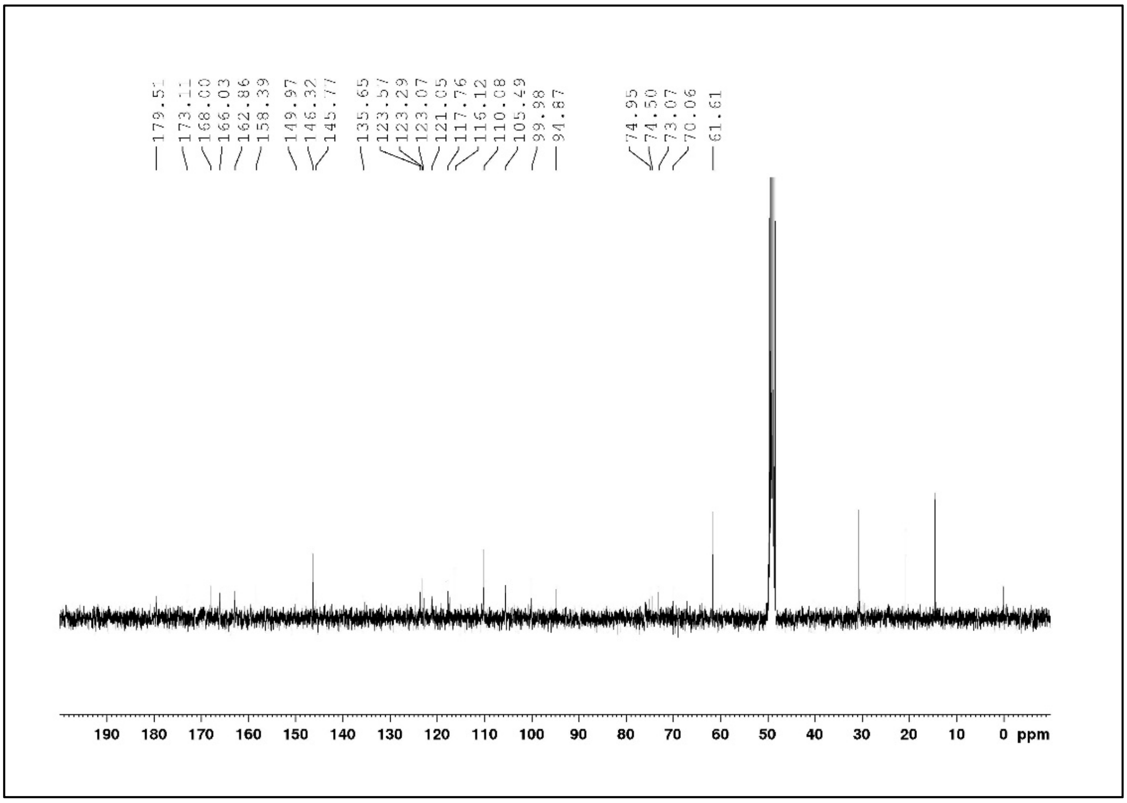

Figure S2. – ^{13}C NMR spectrum of Quercetin-3-*O*-hexosylgalate (**1**) (100 Hz, CD_3OD, δ).

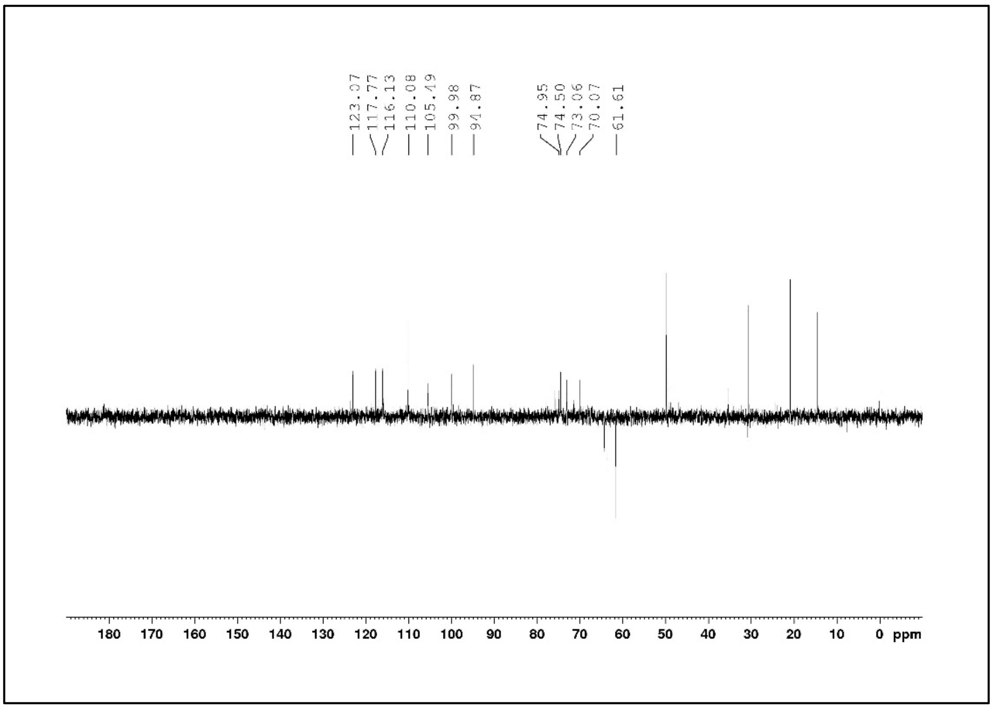

Figure S3. – DEPT-135° NMR spectrum of Quercetin-3-*O*-hexosylgalate (**1**) (100 Hz, CD_3OD, δ).

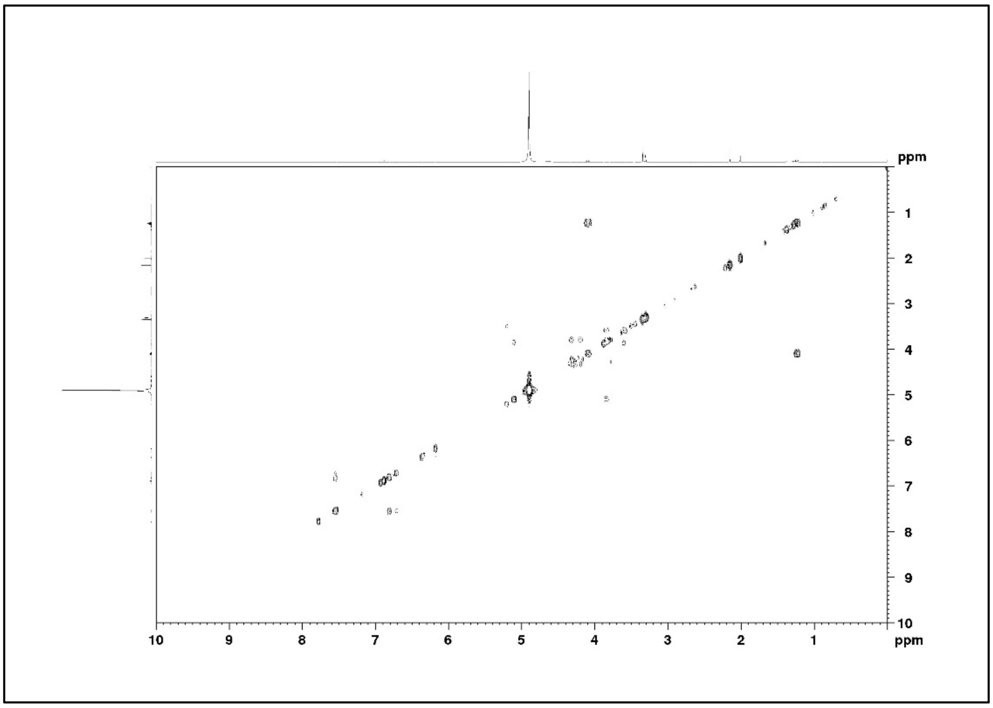

Figure S4.–COSY contour map of Quercetin-3-*O*-hexosylgalate (**1**) (400 Hz, CD_3OD, δ).

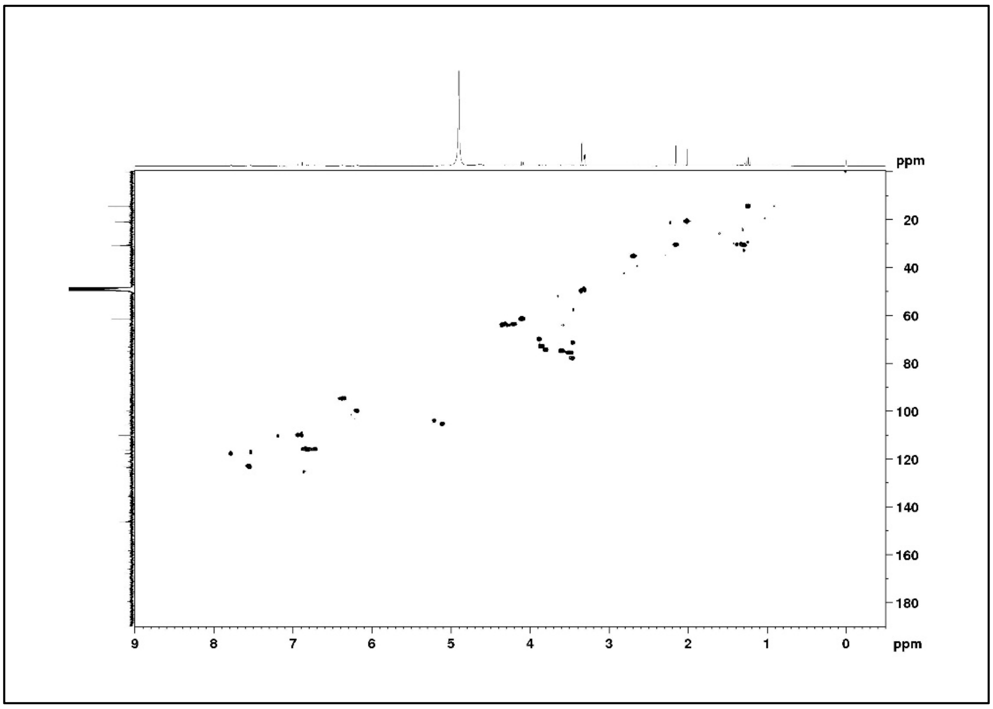

Figure S5.–HSQC contour map of Quercetin-3-*O*-hexosylgalate (**1**) (400 Hz, CD_3OD, δ).

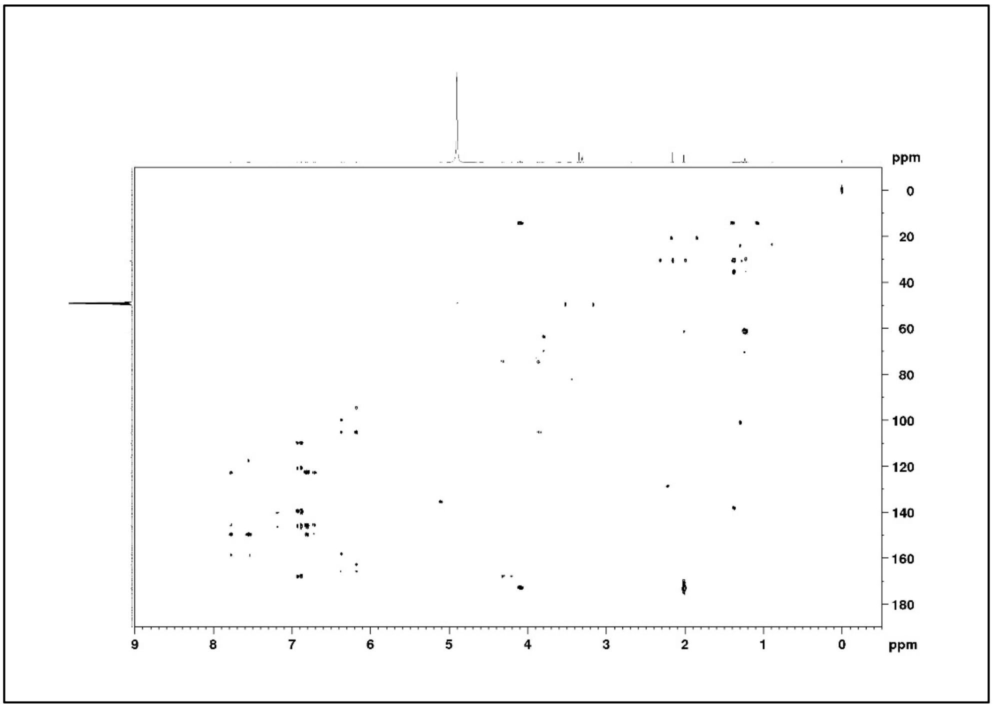

Figure S6. – HMBC contour map of Quercetin-3-*O*-hexosylgalate (**1**) (400 Hz, CD_3OD, δ).

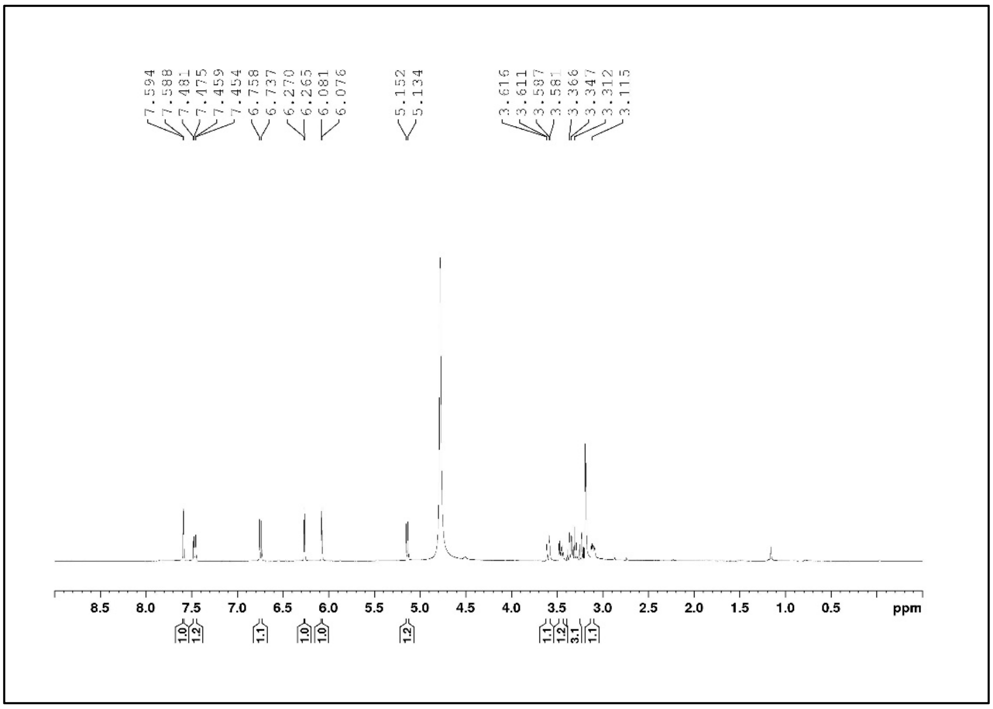

Figure S7. – ^{1}H NMR spectrum of Quercetin-3-O-glucoside (**2**) (400 Hz, CD_3OD, δ).

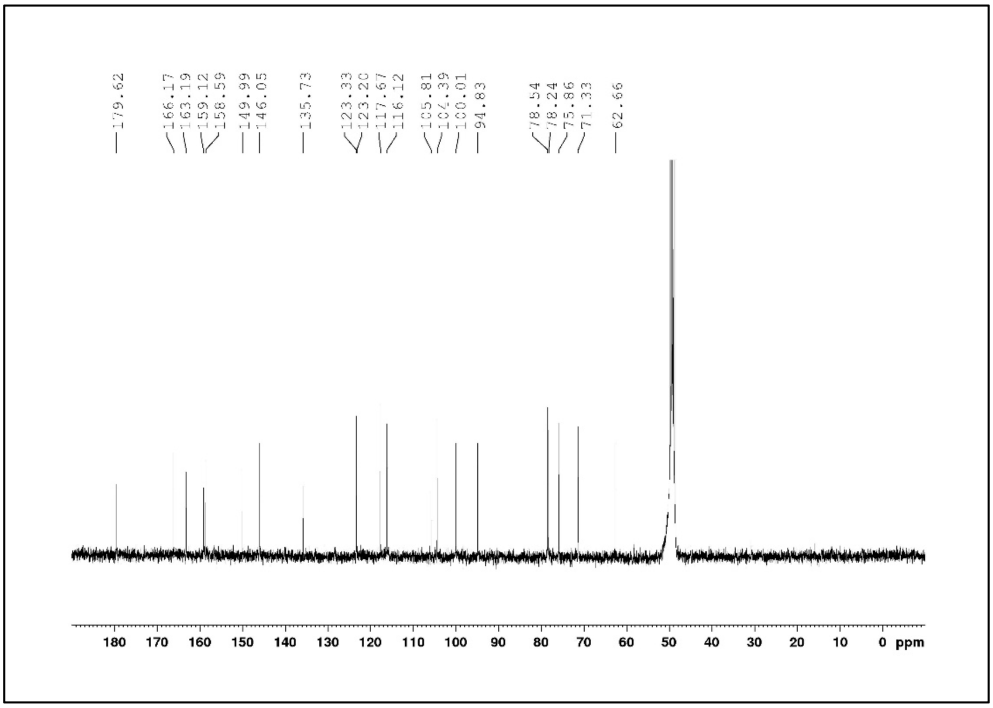

Figure S8. – ^{13}C NMR spectrum of Quercetin-3-O-glucoside (**2**) (100 Hz, CD_3OD, δ).

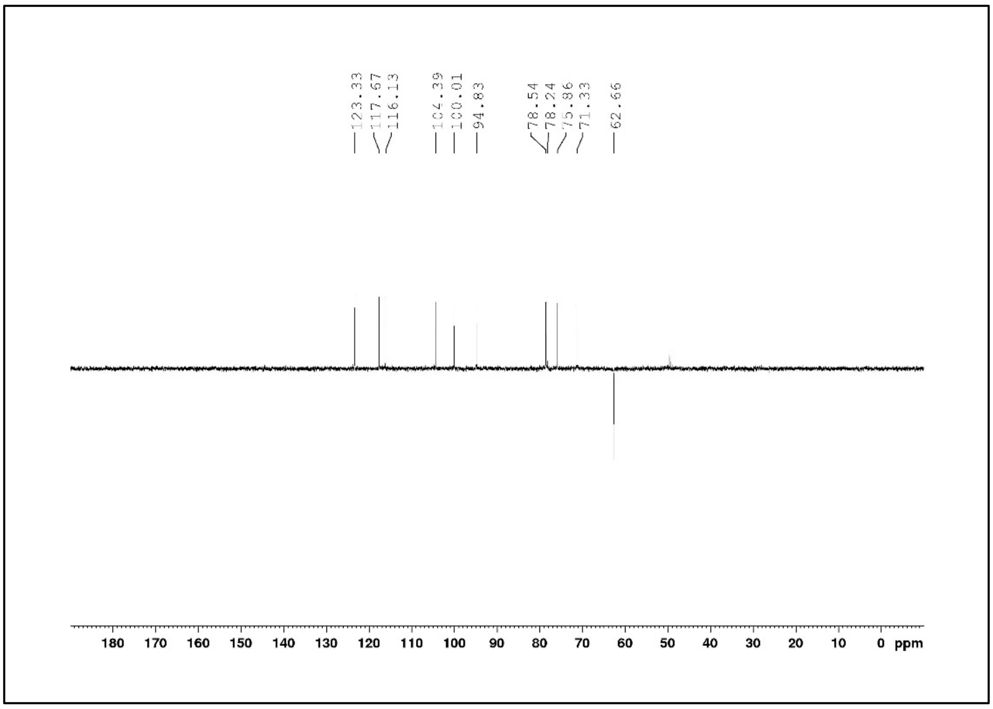

Figure S9. – DEPT-135° NMR spectrum of Quercetin-3-O-glucoside (**2**) (100 Hz, CD_3OD, δ).

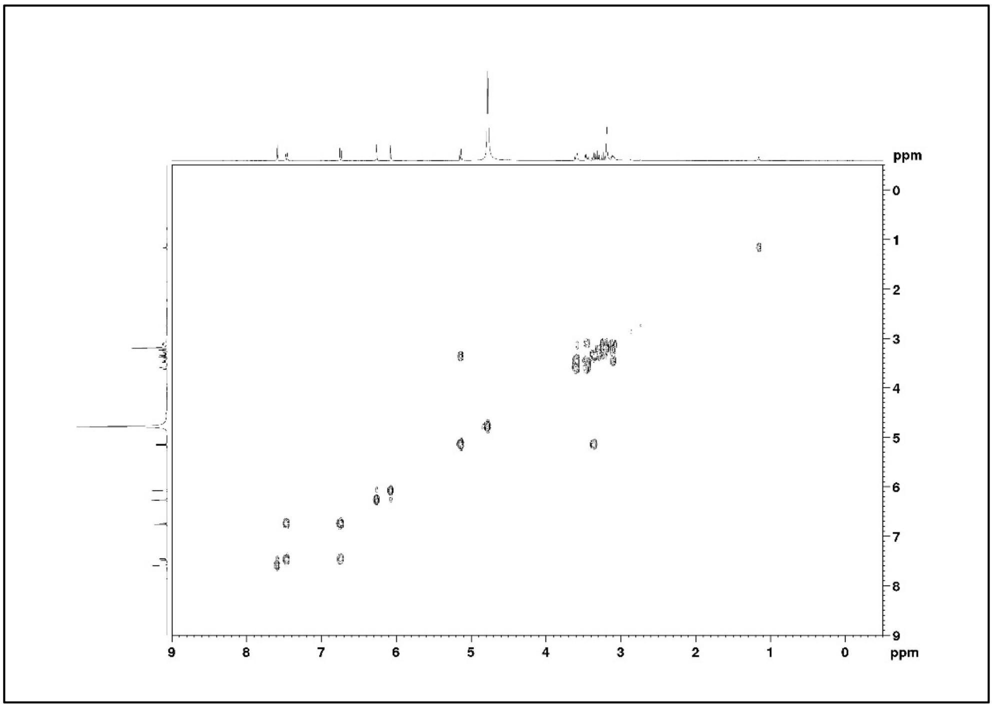

Figure S10. – COSY contour map of Quercetin-3-O-glucoside (**2**) (400 Hz, CD_3OD, δ).

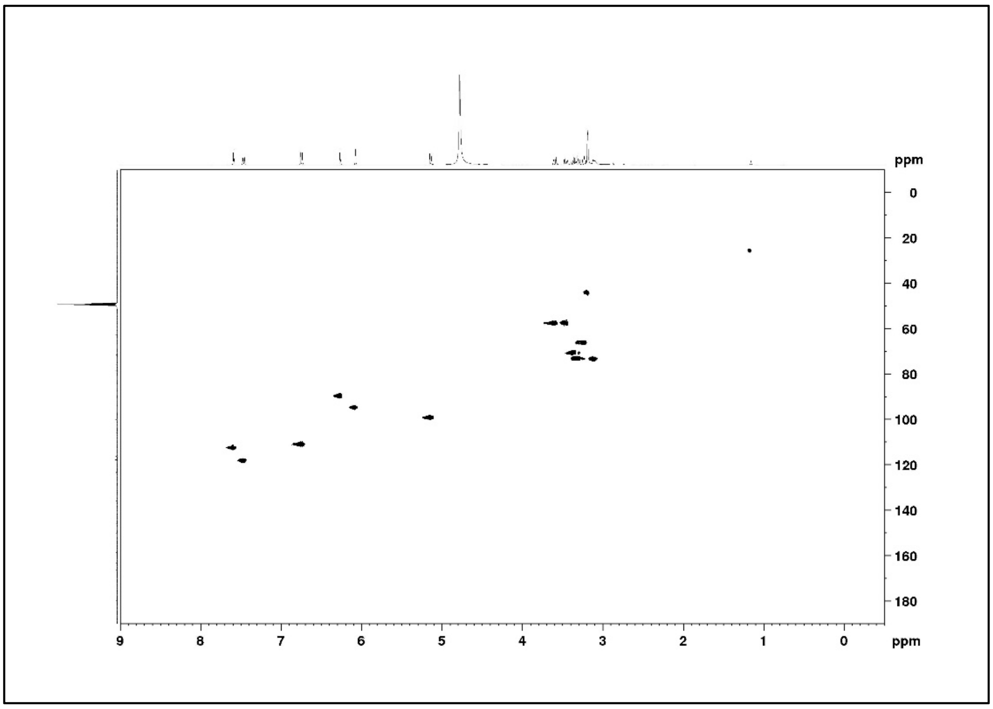

Figure S11. – HSQC contour map of Quercetin-3-O-glucoside (**2**) (400 Hz, CD_3OD, δ).

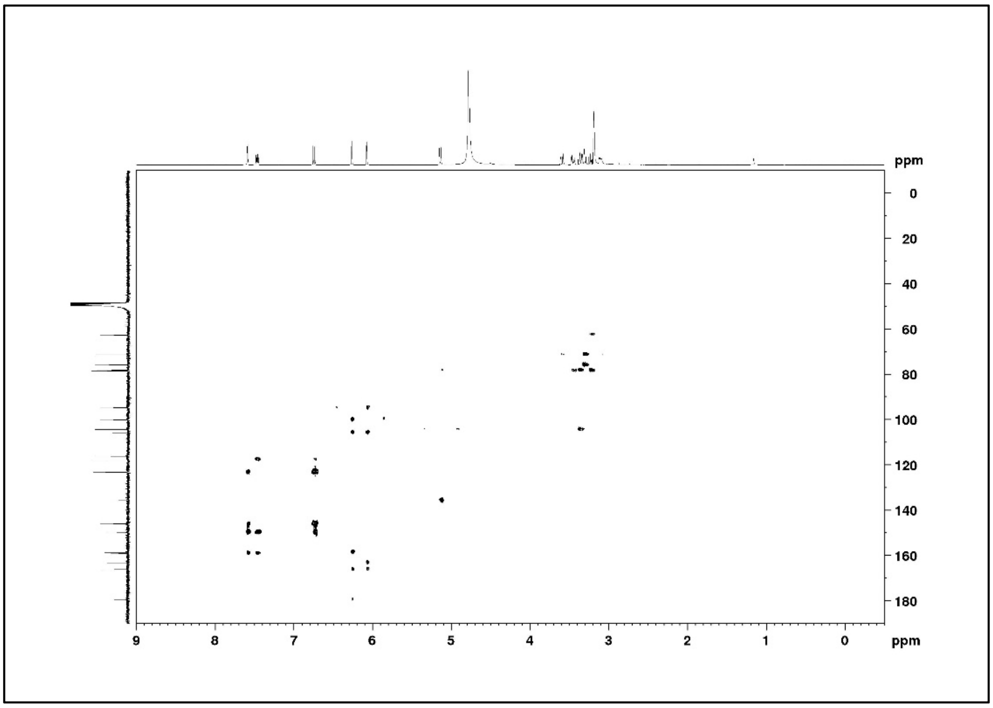

Figure S12. – HMBC contour map of Quercetin-3-O-glucoside (**2**) (400 Hz, CD_3OD, δ).

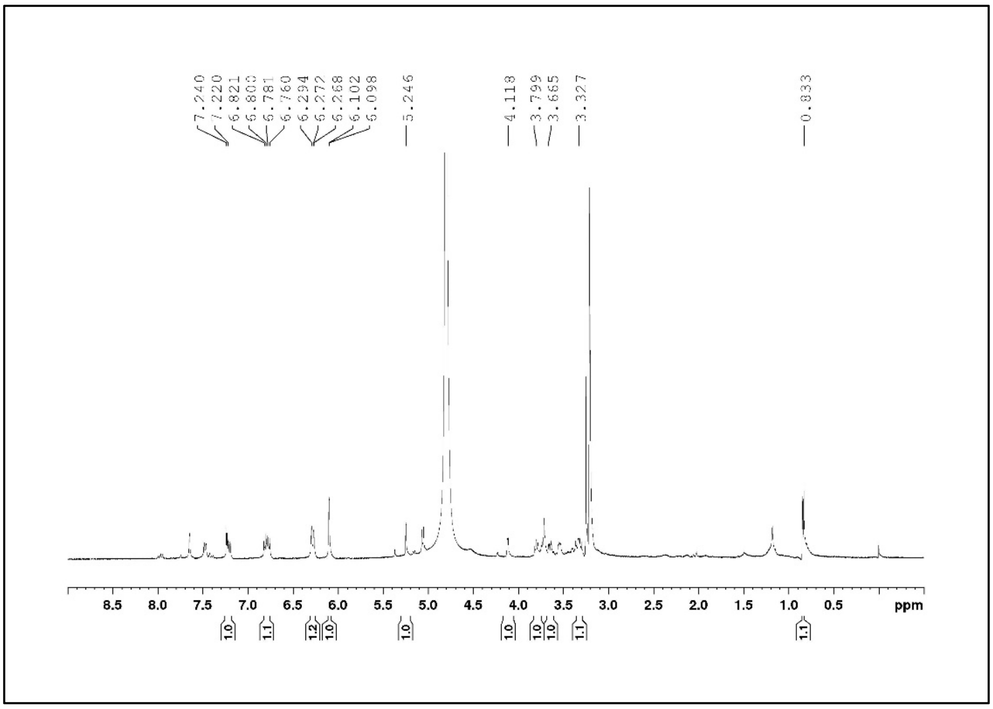

Figure S13. – ^{1}H NMR spectrum of Quercetin-3-O- rhamnoside (**3**) (400 Hz, CD_3OD, δ).

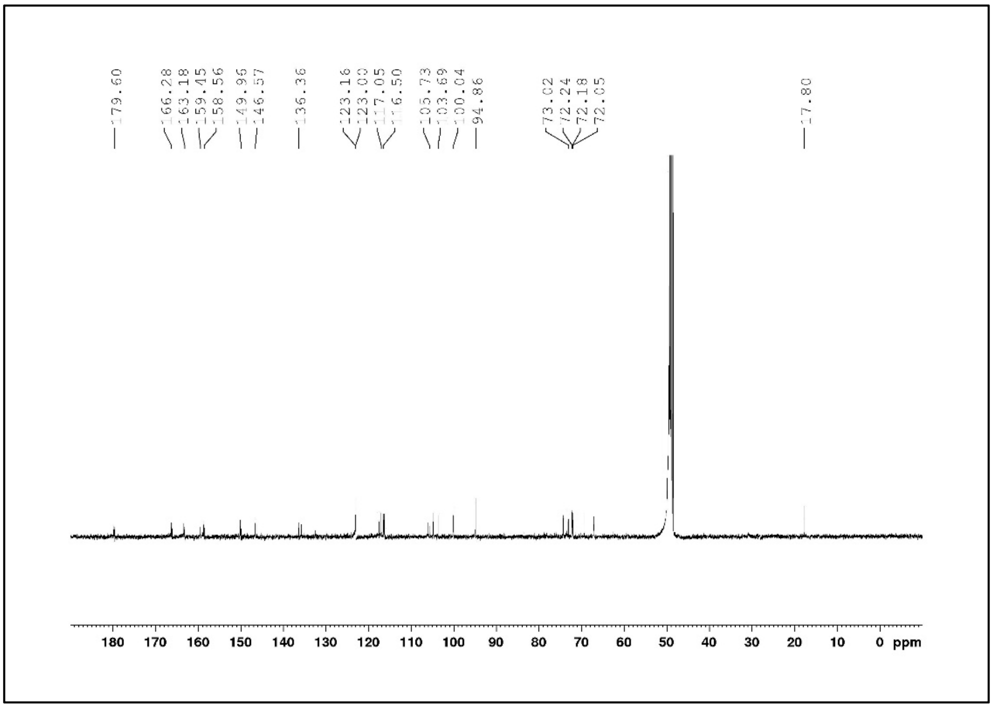

Figure S14. – ^{13}C NMR spectrum of Quercetin-3-O- rhamnoside (**3**) (100 Hz, CD_3OD, δ).

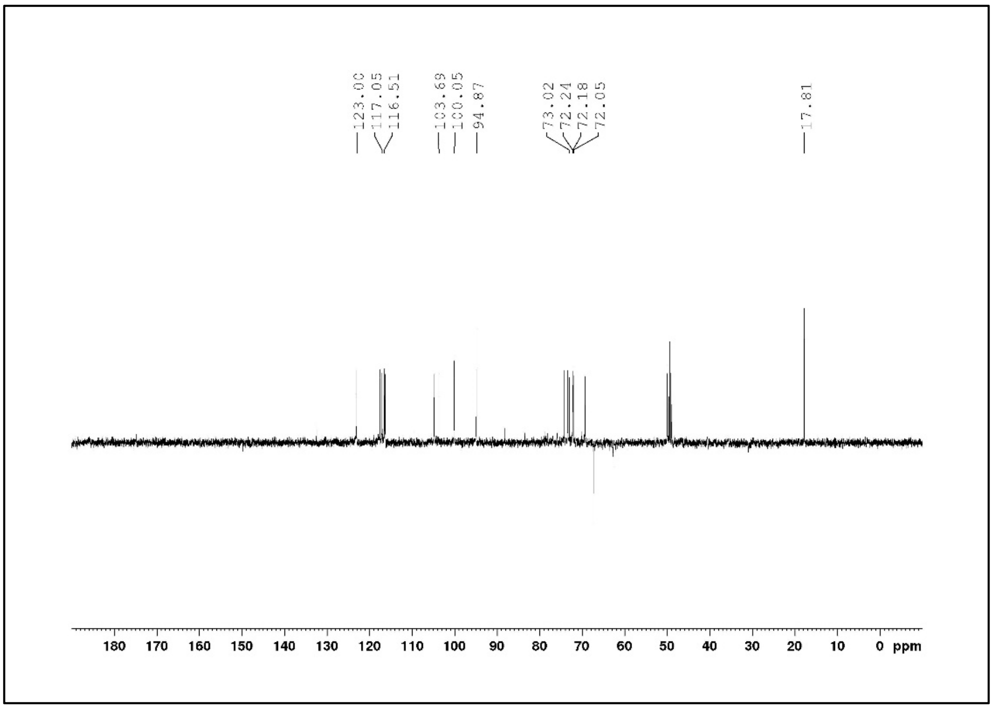

Figure S15. – DEPT-135° NMR spectrum of Quercetin-3-O- rhamnoside (**3**) (100 Hz, CD_3OD, δ).

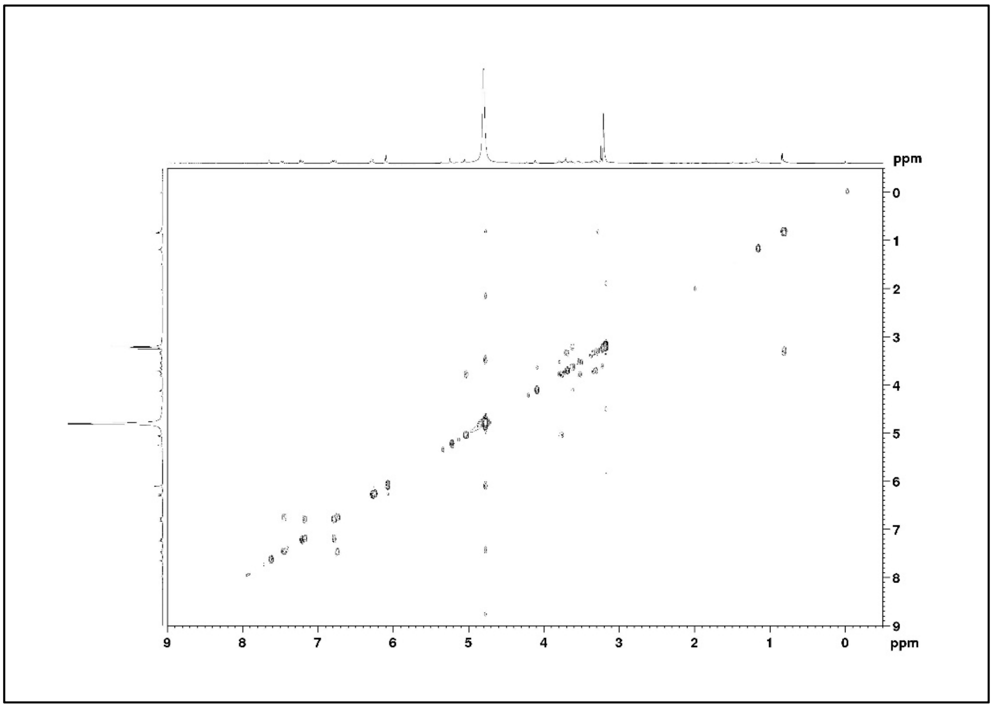

Figure S16. – COSY contour map of Quercetin-3-O- rhamnoside (**3**) (400 Hz, CD_3OD, δ).

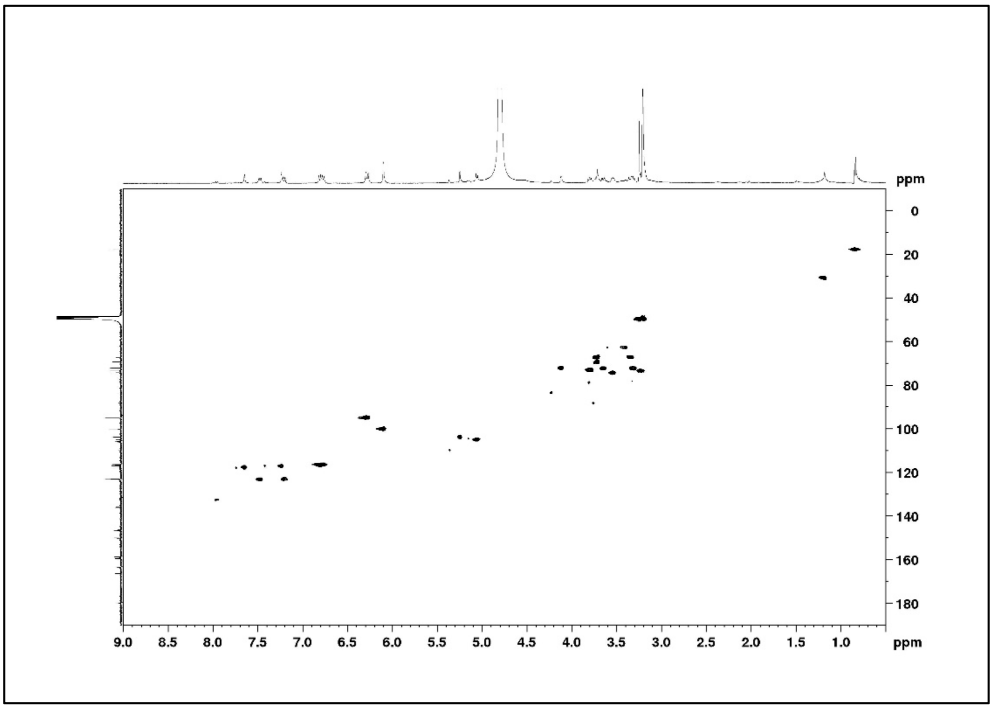

Figure S17. – HSQC contour map of Quercetin-3-O- rhamnoside (**3**) (400 Hz, CD_3OD, δ).

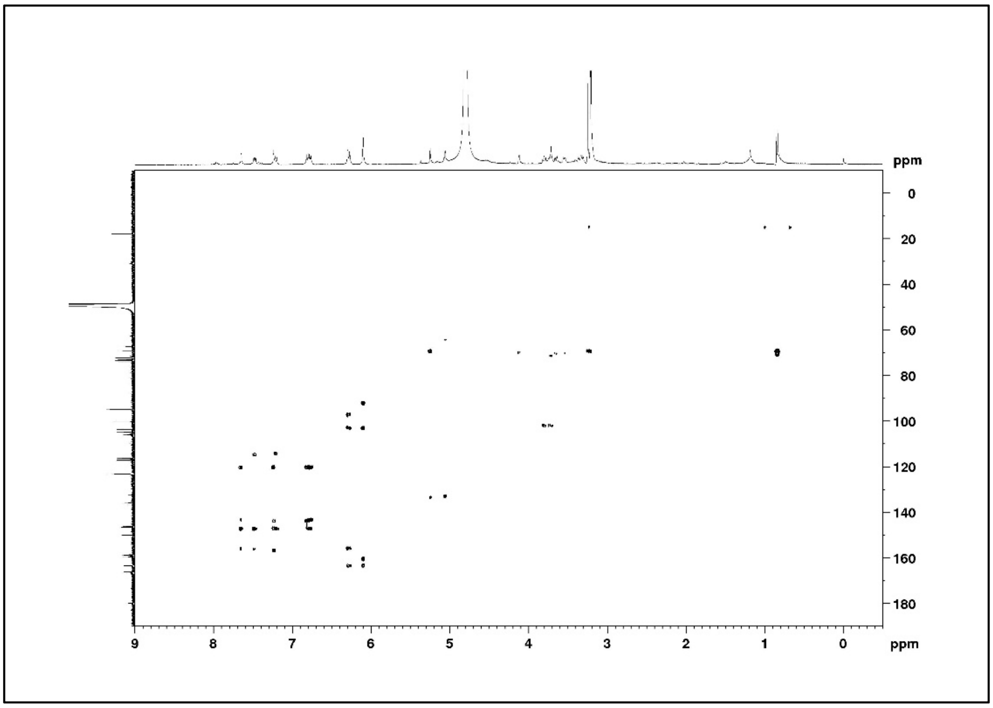

Figure S18. – HMBC contour map of Quercetin-3-O- rhamnoside (**3)** (400 Hz, CD_3OD, δ).

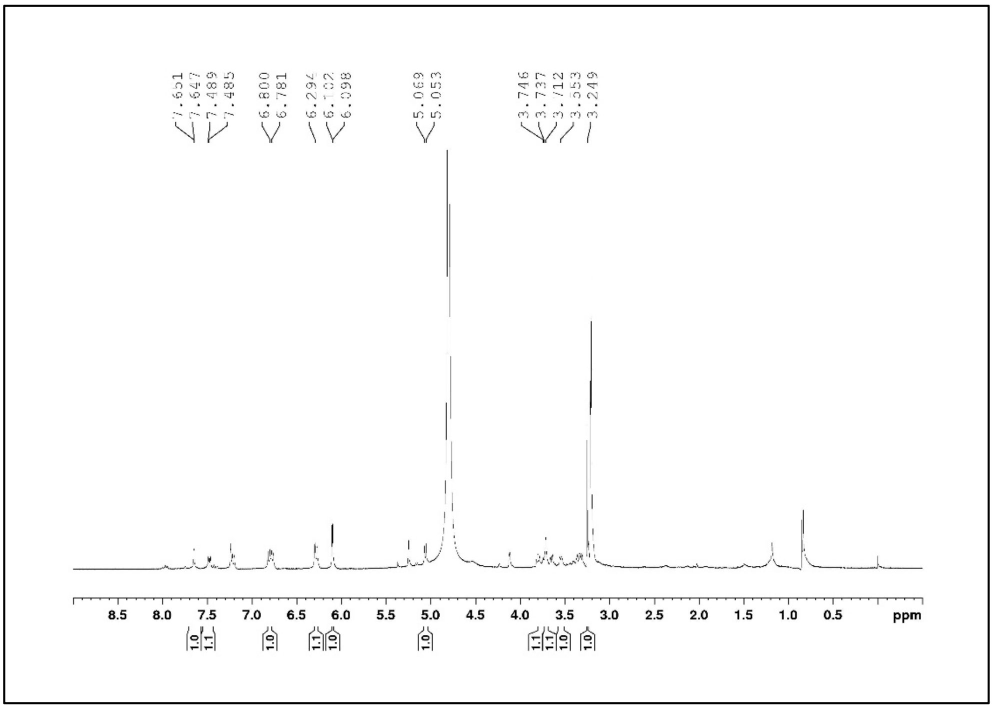

Figure S19. – ^{1}H NMR spectrum of Quercetin-3-O- arabinoside (**4)** (400 Hz, CD_3OD, δ).

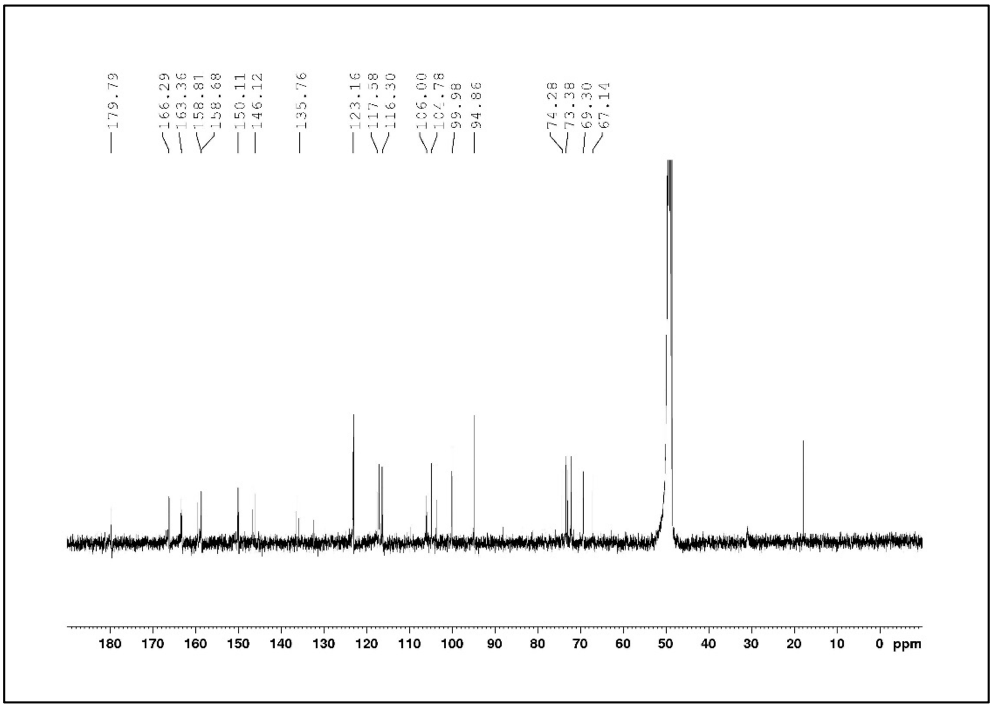

Figure S20. – ^{13}C NMR spectrum of Quercetin-3-O- arabinoside (**4**) (100 Hz, CD_3OD, δ).

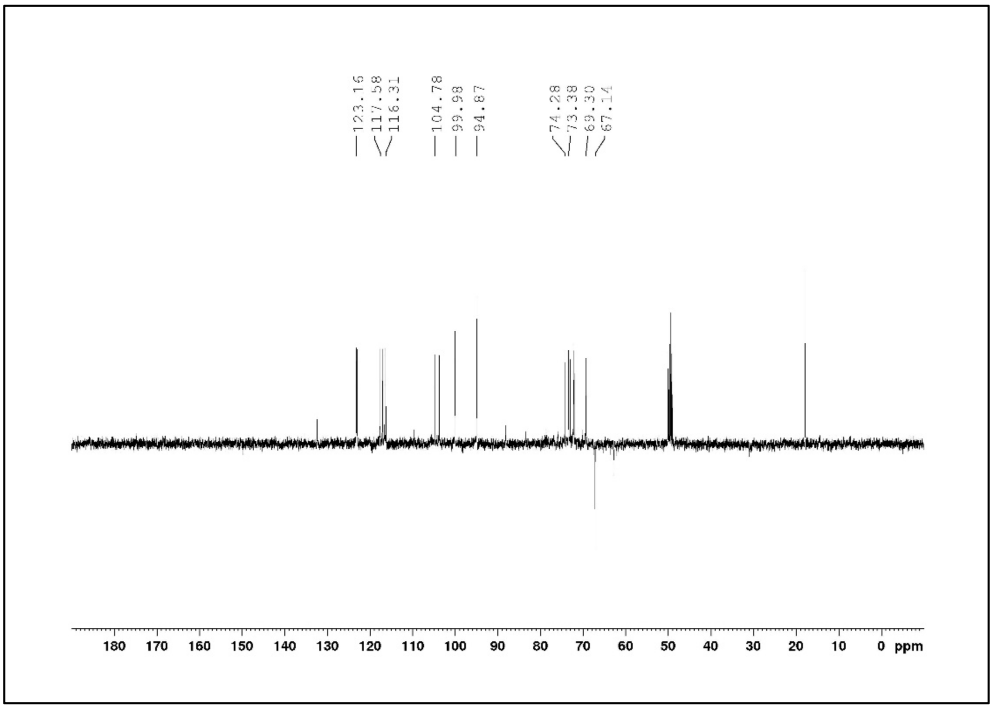

Figure S21. – DEPT-135° NMR spectrum of Quercetin-3-O- arabinoside (**4**) (100 Hz, CD_3OD, δ).

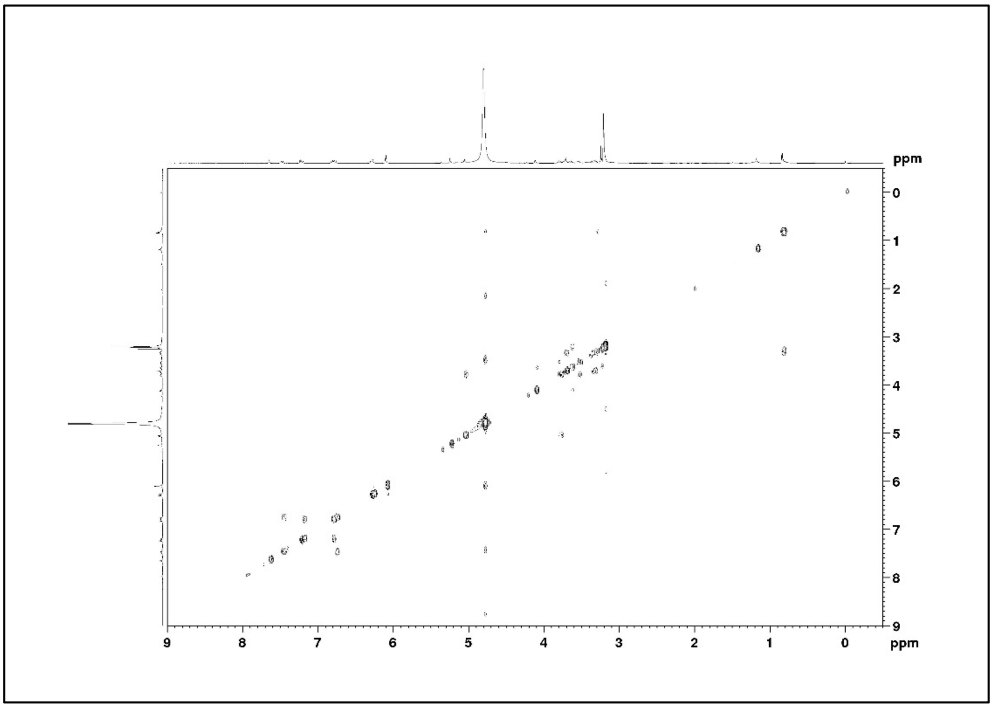

Figure S22. – COSY contour map of Quercetin-3-O- arabinoside (**4**) (400 Hz, CD_3OD, δ).

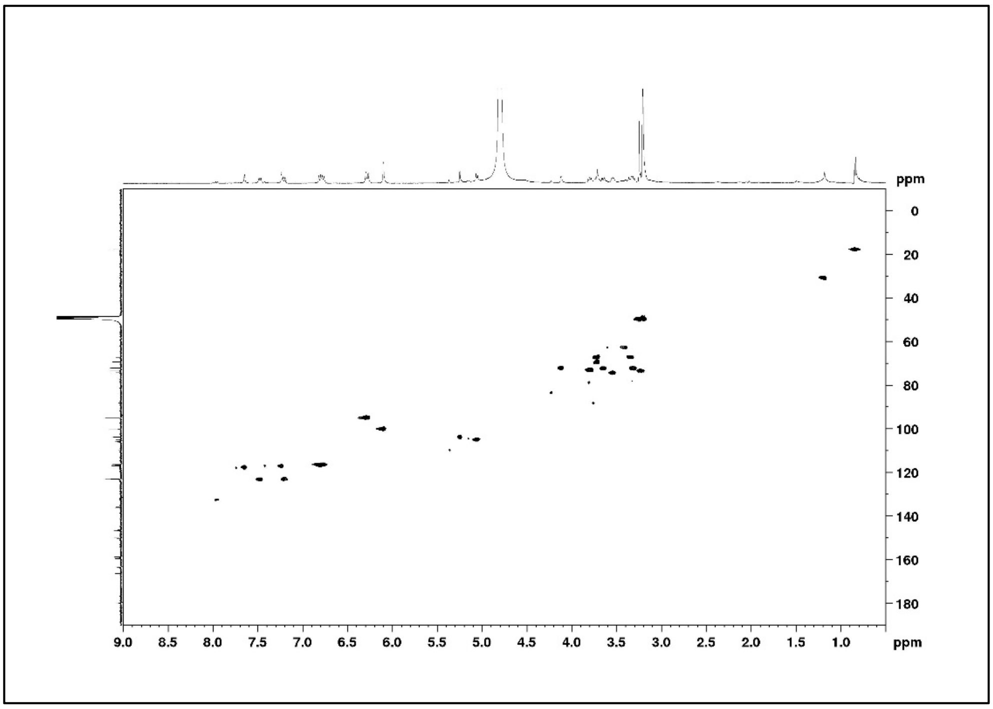

Figure S23. – HSQC contour map of Quercetin-3-O- arabinoside (**4**) (400 Hz, CD_3OD, δ).

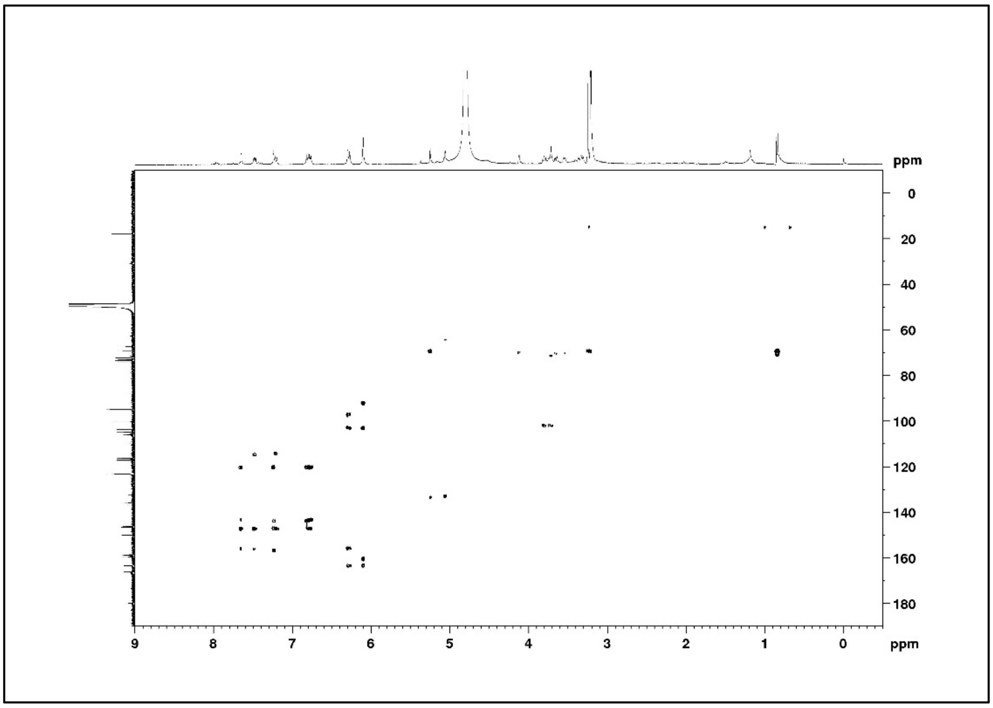

Figure S24. – HMBC contour map of Quercetin-3-O- arabinoside (**4**) (400 Hz, CD3OD, δ).

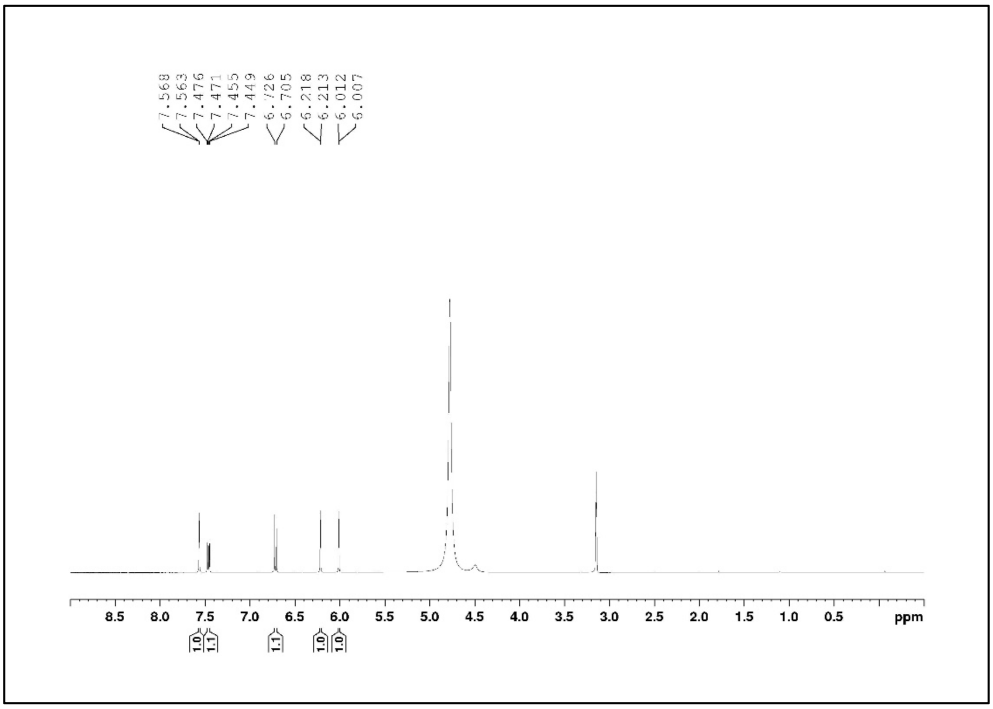

Figure S25. – ^{1}H NMR spectrum of Quercetin (**5**) (400 Hz, CD3OD*6*, δ).

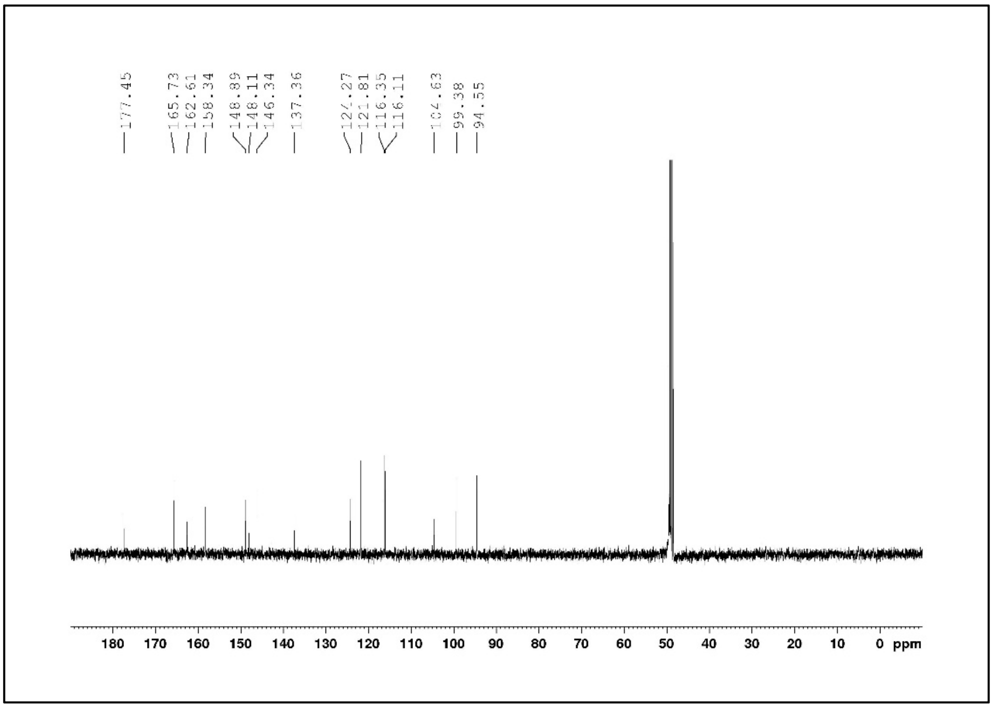

Figure S26. – ^{13}C NMR spectrum of Quercetin (**5**) (100 Hz, CD_3OD, δ).

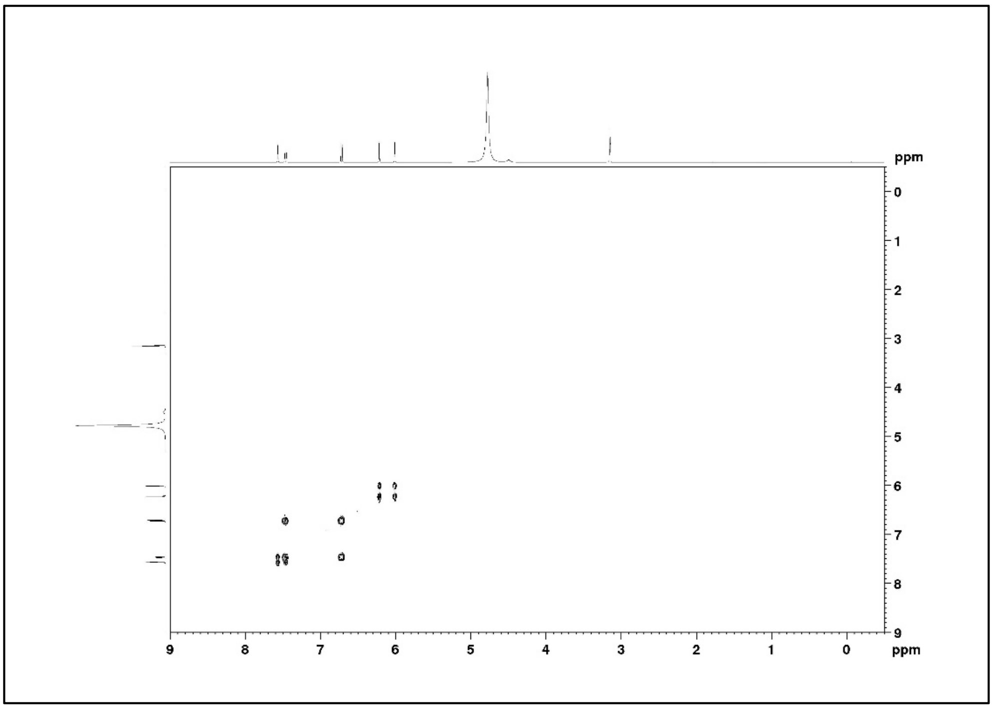

Figure S27. – COSY contour map of Quercetin (**5**) (400 Hz, CD_3OD, δ).

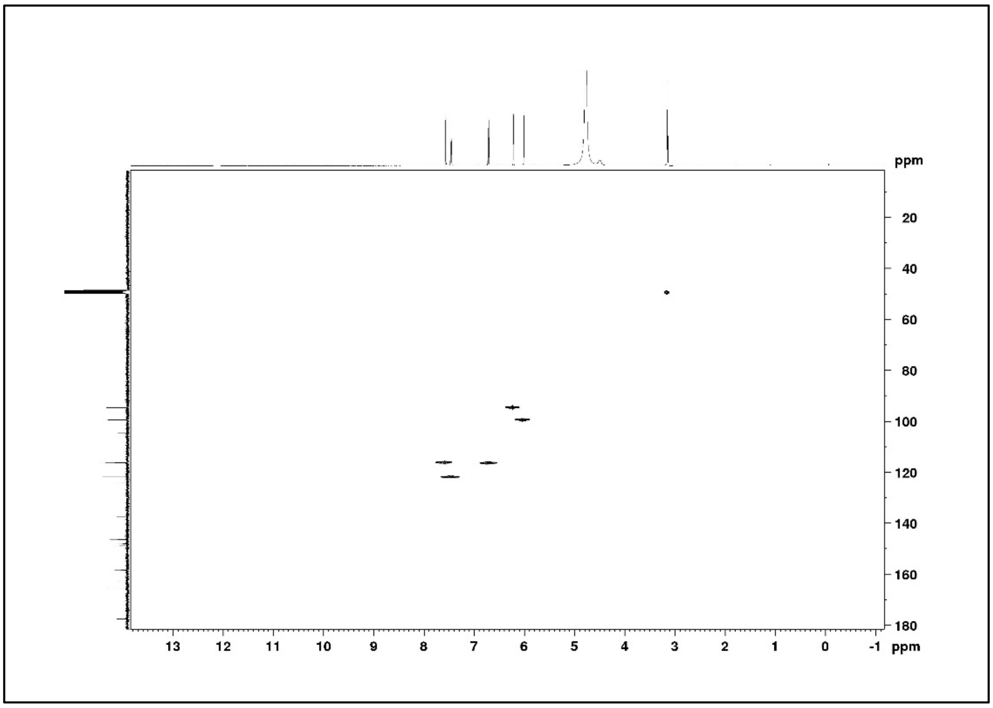

Figure S28. – HSQC contour map of Quercetin (**5)** (400 Hz, CD3OD, δ).

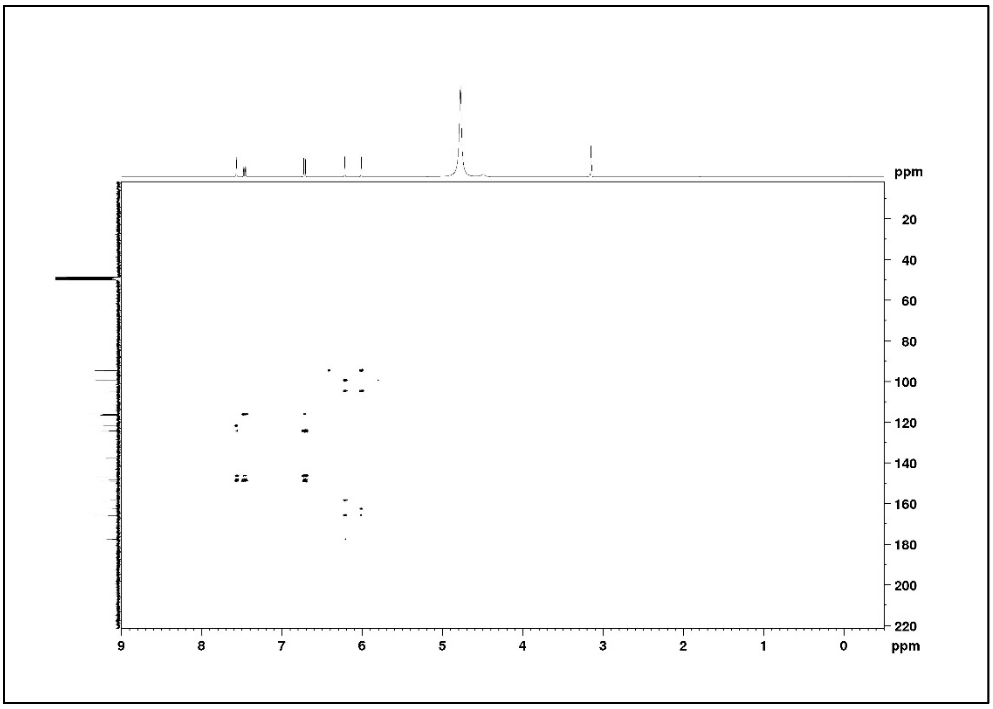

Figure S29. – HMBC contour map of Quercetin (**5)** (400 Hz, CD3OD, δ).
